# Activating Transcription Factor 6 Is Necessary and Sufficient for Alcoholic Fatty Liver Disease in Zebrafish

**DOI:** 10.1371/journal.pgen.1004335

**Published:** 2014-05-29

**Authors:** Deanna L. Howarth, Claudia Lindtner, Ana M. Vacaru, Ravi Sachidanandam, Orkhontuya Tsedensodnom, Taisa Vasilkova, Christoph Buettner, Kirsten C. Sadler

**Affiliations:** 1 Department of Medicine – Division of Liver Diseases, Icahn School of Medicine at Mount Sinai, New York, New York, United States of America; 2 Department of Developmental and Regenerative Biology, Icahn School of Medicine at Mount Sinai, New York, New York, United States of America; 3 Department of Medicine – Division of Endocrinology, Diabetes, and Bone Disease, Icahn School of Medicine at Mount Sinai, New York, New York, United States of America; 4 Diabetes and Metabolism Institute, Icahn School of Medicine at Mount Sinai, New York, New York, United States of America; 5 Department of Genetics and Genomic Sciences, Icahn School of Medicine at Mount Sinai, New York, New York, United States of America; 6 Graduate School of Biomedical Sciences, Icahn School of Medicine at Mount Sinai, New York, New York, United States of America; University of Pennsylvania School of Medicine, United States of America

## Abstract

Fatty liver disease (FLD) is characterized by lipid accumulation in hepatocytes and is accompanied by secretory pathway dysfunction, resulting in induction of the unfolded protein response (UPR). Activating transcription factor 6 (ATF6), one of three main UPR sensors, functions to both promote FLD during acute stress and reduce FLD during chronic stress. There is little mechanistic understanding of how ATF6, or any other UPR factor, regulates hepatic lipid metabolism to cause disease. We addressed this using zebrafish genetics and biochemical analyses and demonstrate that Atf6 is necessary and sufficient for FLD. *atf6* transcription is significantly upregulated in the liver of zebrafish with alcoholic FLD and morpholino-mediated *atf6* depletion significantly reduced steatosis incidence caused by alcohol. Moreover, overexpression of active, nuclear Atf6 (nAtf6) in hepatocytes caused FLD in the absence of stress. mRNA-Seq and qPCR analyses of livers from five day old nAtf6 transgenic larvae revealed upregulation of genes promoting glyceroneogenesis and fatty acid elongation, including fatty acid synthase (*fasn*), and nAtf6 overexpression in both zebrafish larvae and human hepatoma cells increased the incorporation of ^14^C-acetate into lipids. Srebp transcription factors are key regulators of lipogenic enzymes, but reducing Srebp activation by *scap* morpholino injection neither prevented FLD in nAtf6 transgenics nor synergized with *atf6* knockdown to reduce alcohol-induced FLD. In contrast, *fasn* morpholino injection reduced FLD in nAtf6 transgenic larvae and synergistically interacted with *atf6* to reduce alcoholic FLD. Thus, our data demonstrate that Atf6 is required for alcoholic FLD and epistatically interacts with *fasn* to cause this disease, suggesting triglyceride biogenesis as the mechanism of UPR induced FLD.

## Author Summary

Fatty liver disease (steatosis) is the most common liver disease worldwide and is commonly caused by obesity, type 2 diabetes, or alcohol abuse. All of these conditions are associated with impaired hepatocyte protein secretion, resulting in hypoproteinemia that contributes to the systemic complications of these diseases. The unfolded protein response (UPR) is activated in response to stress in the protein secretory pathway and a wealth of data indicates that UPR activation can contribute to steatosis, but the mechanistic basis for this relationship is poorly understood. We identify activating transcription factor 6 (Atf6), one of three UPR sensors, as necessary and sufficient for steatosis and show that Atf6 activation can promote lipogenesis, providing a direct connection between the stress response and lipid metabolism. Blocking Atf6 in zebrafish larvae prevents alcohol-induced steatosis and Atf6 overexpression in zebrafish hepatocytes induces genes that drive lipogenesis, increases lipid production and causes steatosis. Fatty acid synthase (*fasn*) is a key lipogenic enzyme and we show that *fasn* is required for fatty liver in response to both ethanol and Atf6 overexpression. Our findings point to Atf6 as a potential therapeutic target for fatty liver disease.

## Introduction

The unfolded protein response (UPR) acts in most cells to maintain homeostasis within the protein secretory pathway during physiological conditions. During stress, the UPR becomes further induced to mitigate the accumulation of misfolded or unfolded proteins in the endoplasmic reticulum (ER). If UPR activation cannot relieve the excess protein load, the ER becomes dilated and dysfunctional, and all branches of the UPR remain chronically activated in a condition referred to as ER stress. Many studies have implicated ER stress in a range of pathologies, and there is a clear association between UPR activation and metabolic diseases such as fatty liver disease (FLD) [1]–[5]. However, it is not known whether factors that control the UPR can also directly impact lipid metabolism and it remains unclear how UPR activation causes FLD.

FLD is the most common hepatic pathology worldwide [6], and alcohol abuse is a leading cause. Even a single episode of binge drinking causes lipid accumulation in hepatocytes (steatosis) in over 90% of drinkers [7]. While acute steatosis can resolve, chronic steatosis can render hepatocytes susceptible to damage and is a prerequisite step in developing more severe liver disease, including steatohepatitis and cirrhosis. Most FLD etiologies are accompanied by impairment of protein secretion by hepatocytes that result in serum protein deficiencies, which are most apparent in chronic alcoholics and contribute to the systemic complications of alcoholic liver disease (ALD). These defects are reflected in many reports demonstrating that alcohol induces some UPR sensors and targets in the liver of mice [1], [8], rats [9], micropigs [10], and zebrafish [11]–[13]. Moreover, deleting ATF4 or CHOP, two key UPR effectors, reduces ethanol-induced liver injury [8], [14], indicating that UPR activation can contribute to ALD. However, it is not clear whether these genes, or other UPR effectors, contribute to steatosis.

There are three main UPR sensors that function through shared and independent mechanisms to maintain ER function by enhancing protein processing and folding in the ER, and by promoting degradation of terminally misfolded secretory proteins. Activating transcription factor 6 (ATF6) and inositol-requiring enzyme 1-alpha (IRE1α or ERN1) pathways generate the active transcription factors nATF6 and XBP1, respectively, that induce hundreds of UPR target genes [15], [16]. PRKR-like endoplasmic reticulum kinase (PERK or EIF2AK3) phosphorylates EIF2A to repress translation [17] and to promote production of ATF4 [18], which induces a subset of target genes [14], [18]. Each of these primary sensors has been evaluated for its contribution to FLD caused by a robust ER stressor [19], [20]. ATF6 knockout mice fail to resolve steatosis caused by acute stress due to tunicamycin injection [16], [21], [22], suggesting that loss of ATF6 promotes fatty liver. Our previous work using zebrafish confirmed the finding that steatosis caused by acute stress is augmented by *atf6* loss, and also demonstrates that steatosis caused by chronic stress is reduced when Atf6 is depleted [23]. Thus, it appears that *atf6* loss can alternatively enhance or reduce FLD, depending on the nature and duration of the stress.

The relationship between metabolic disease and UPR activation is under intensive investigation, yet several important questions remain unanswered. First, is UPR activation a cause or consequence of FLD? While some studies indicate that lipotoxicity and fatty acid accumulation can induce the UPR [24], [25], there is incontrovertible evidence that robust UPR activation is sufficient to induce steatosis [2], [26], [27]. These data are incorporated into a current model proposing that robust UPR activation can cause steatosis and, if the lipid burden is not resolved, this can further augment cellular stress and contribute to chronic UPR induction. The second question is which, if any, of the main UPR factors directly cause fatty liver? All studies to date that have addressed this question, including our work in zebrafish [23], utilize loss of function approaches whereby a key UPR gene is deleted and the effect on stress-induced steatosis is evaluated. However, these studies are complicated by the extensive crosstalk between UPR effectors, and by the ability of cells to adapt to changes in UPR capacity. Third, what is the mechanism by which UPR activation causes fatty liver? Activation of lipogenic genes by the sterol response element binding protein (SREBP) transcription factors has been implicated as an essential pathway in both alcoholic [28], [29] and non-alcoholic [30]–[32] FLD. Since SREBPs and ATF6 are activated by a shared proteolytic mechanism [33], it is possible that these two proteins are activated simultaneously in response to stress. However, the role of SREBPs in FLD caused by UPR activation has not been addressed.

Here, we use genetic and genomic approaches in a well-established zebrafish model of ALD [11]–[13], [34]–[37] to demonstrate that blocking Atf6 prevents alcohol-induced steatosis and that overexpression of the nuclear, active form of Atf6 (nAtf6) causes steatosis. We found that nAtf6 overexpression increases expression of genes in the lipogenic pathway and alters lipid flux to promote triglyceride synthesis by a mechanism that does not involve Srebps. Instead, we demonstrate an epistatic interaction between *atf6* and *fasn* in driving alcoholic steatosis. These findings demonstrate the first clear and causative link between a central UPR sensor and FLD.

## Results

### Atf6 is required for alcoholic steatosis

**Figure 1:**
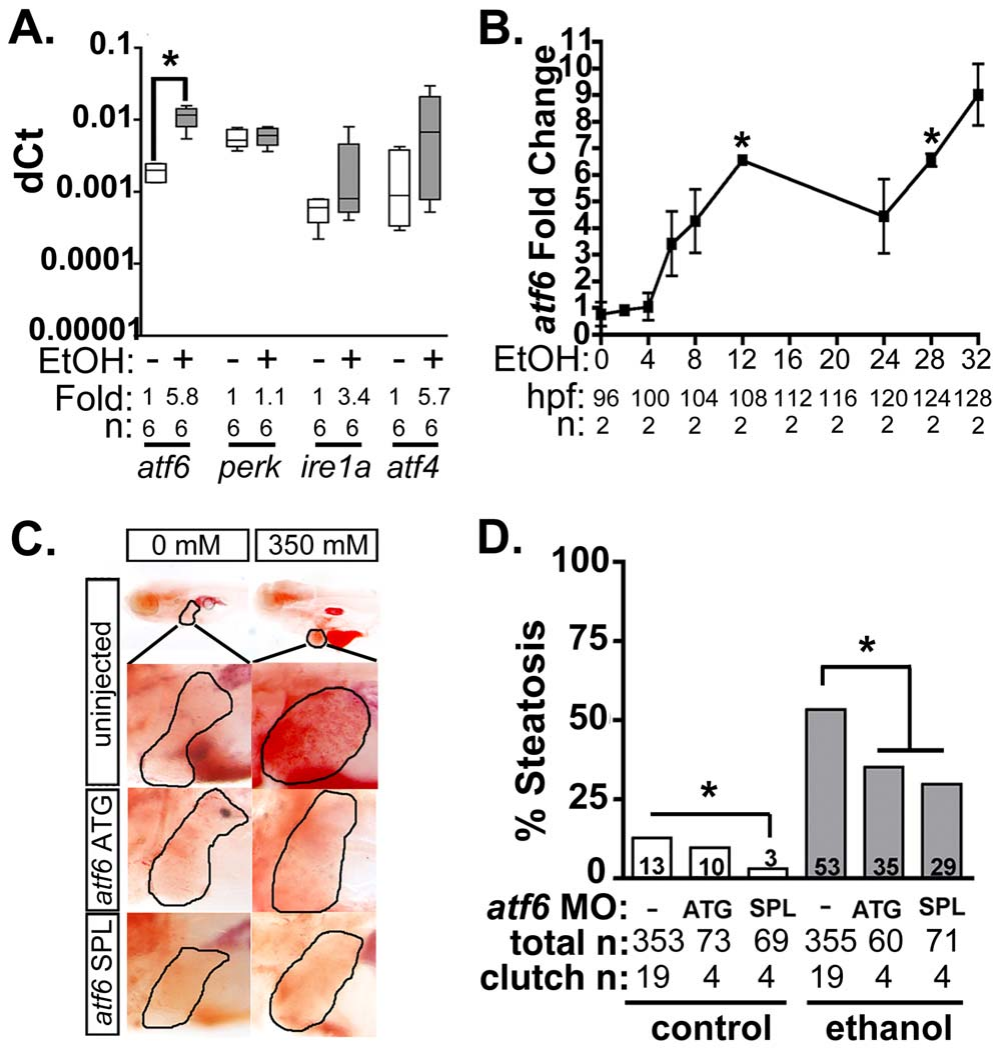
Atf6 is required for alcoholic steatosis. **A:** qPCR analysis of UPR sensors *atf6, perk, ire1a,* and *atf4* in the livers of control (EtOH-) and 350 mM ethanol-treated (EtOH+) larvae. dCt values were calculated by normalization to *rpp0.* Fold changes were calculated based on median dCt values. Statistics: paired t-test. *, p<0.05. **B:** Expression of *atf6* in the livers of 350 mM ethanol treated larvae from 0–32 hours. Fold changes were determined by normalizing to control (0 mM EtOH) dCt values. Statistics: one-sample t-test. *, p<0.05. **C:** Images of oil red O stained whole larvae injected with two different morpholinos targeting *atf6* (ATG-targeting, *atf6* ATG, and splice-blocking, *atf6* SPL) and exposed to either 0 or 350 mM ethanol. Livers are circled in the enlarged boxes. **D:** Steatosis incidence based on scoring oil red O stained larvae at 5.5 dpf. Statistics: chi-square with Fisher's Exact Test. *, p<0.05. “Total n” and “clutch n” corresponds to the number of larvae and number of clutches scored.

We previously reported that transcription of multiple UPR effector genes and translation of the major ER chaperone, Bip, are induced in zebrafish livers following alcohol exposure [11]–[13]. We have also demonstrated that *xbp1* splicing, a direct measure of Ire1a activation, is only detectable within the first few hours of exposure to ethanol [13] yet UPR target gene induction persists long afterwards [12], suggesting that the Ire1a pathway is not entirely responsible for the UPR in ALD. Our previous finding that Eif2a is phosphorylated in the liver of zebrafish with ALD [13] suggests that Perk is activated by alcohol, but since Eif2a can also be phosphorylated by other kinases [38], this has yet to be resolved. The lack of reliable antibodies that recognize the active form of Atf6, Perk and Ire1a in zebrafish hampered direct measurement of the activation status of each pathway (not shown). However, quantitative real-time PCR (qPCR) analysis of liver samples from 5 day post fertilization (dpf) larvae treated with alcohol demonstrated that *atf6* mRNA was significantly induced in the liver (Figure 1A) prior to the onset of steatosis (see Figure 1B and [13]). Since Atf6 has been shown to act in a positive feedback loop to induce its own expression [39], these data suggest that the increase in *atf6* expression could reflect Atf6 activation in response to alcohol.

To test whether Atf6 was required for alcoholic steatosis, we injected a morpholino (MO) targeting either the translation initiation site (*atf6*MO-ATG) [23] or the boundary between coding exon 1 and intron 1 of *atf6* (*atf6*MO-SPL) (Table S1 and Figure S1A), which blocked splicing (Figure S1B) and introduced an early stop codon (Figure S1C). Morphants and uninjected controls were treated with 350 mM ethanol for 32 hours, stained with the neutral lipid dye, oil red O and scored for the presence or absence of steatosis (Figure 1C and S2A). Consistent with previous findings [11]–[13], [23], [34], the percent of larvae with steatosis (i.e. steatosis incidence) was significantly higher in uninjected larvae exposed to 350 mM ethanol for 32 hours (13% in untreated larvae vs. 53% in ethanol treated larvae; p<0.05). *atf6* morphants had reduced steatosis incidence (p<0.05, Figure 1C-D), but still displayed other gross morphological abnormalities caused by ethanol including increased liver circularity, an indication of hepatomegaly (Figure S2B-C), a common feature of liver disease. Thus, Atf6 is required for alcoholic steatosis.

### nAtf6 overexpression in hepatocytes induces robust UPR activation

To determine whether Atf6 is sufficient to cause FLD, we created a transgenic zebrafish line expressing the predicted nuclear, active fragment of zebrafish Atf6 (amino acids 1-366; Figure S3A) fused to mCherry under the hepatocyte-specific promoter *fabp10,* along with a cassette that expressed GFP in cardiomyocytes as a marker of transgenesis ((*Tg(fabp10:nAtf6-cherry*; *cmlc2:GFP)* hereafter called nAtf6 TG). There is high sequence identity between human and zebrafish Atf6 proteins in the transmembrane, leucine zipper and basic (bZIP) domains and the protease recognition sites are highly conserved (Figure S3A). To confirm its nuclear localization, the predicted nAtf6 fragment of the zebrafish protein was fused to GFP and transfected into HepG2 cells (Figure S3B).

**Figure 2:**
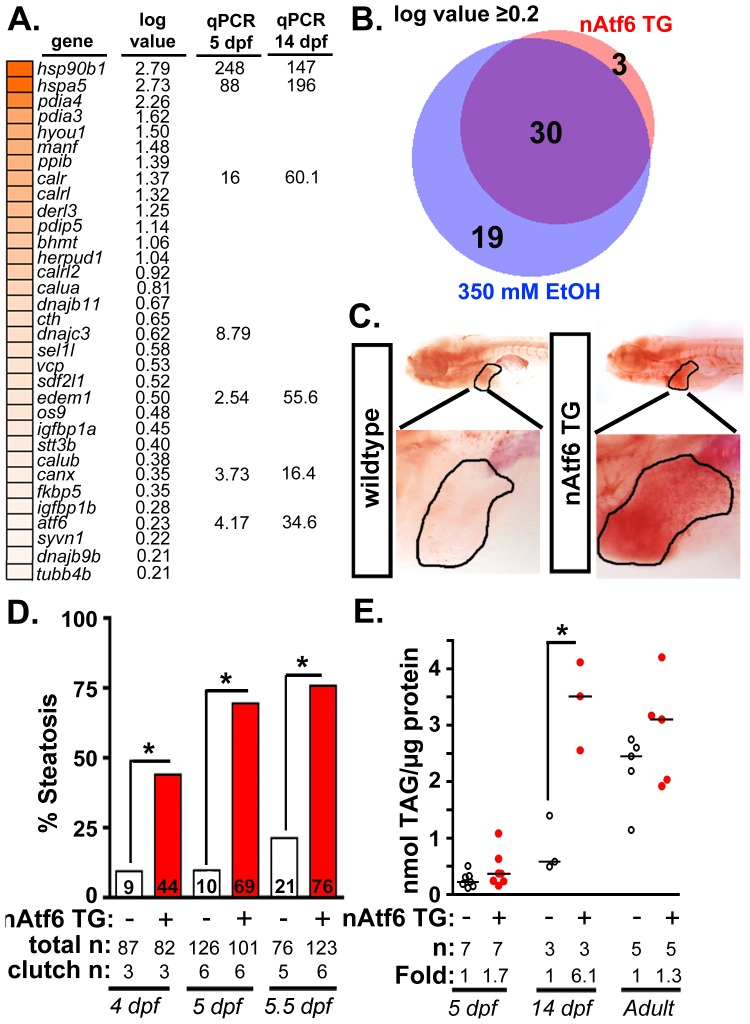
nAtf6 overexpression is sufficient to drive steatosis. **A:** Heatmap of upregulated UPR effector genes in nAtf6 transgenic larvae. The log values based on mRNA-Seq analysis and the median fold change in 3–6 liver samples assessed by qPCR at 5 dpf and 14 dpf. **B:** Most Atf6 targets are upregulated in the liver in response to ethanol. Genes identified as part of the UPR are significantly induced in the liver of nAtf6 TG and ethanol treated larvae (log value ≥ 0.2; see Table S2). **C:** Oil red O stained WT and nAtf6 transgenic larvae. Livers are circled in the enlarged boxes. **D:** Steatosis incidence based on scoring of whole mount oil red O stained larvae at 4, 5, and 5.5 dpf. Statistics: chi-square with Fisher's Exact Test. *, p<0.05. “Total n” and “clutch n” corresponds to the number of larvae and number of clutches scored. **E:** Hepatic triglyceride levels in extracts from pooled livers from WT and nAtf6 transgenic larvae at 5 dpf and 14 dpf, and from single adult livers (∼9 months). Statistics: unpaired t-test. *, p<0.05. Median fold changes are noted.

Transgenics were selected based on GFP expression in the heart, appeared grossly normal throughout development (Figure S3C), and developed into viable, fertile adults. nAtf6-mCherry was not detectable using low-resolution fluorescence microscopy in larvae from all four of the transgenic lines we generated. However, we confirmed transgene expression by PCR and blotting for mCherry (Figures S3D-E). *atf6* mRNA expression in the liver was ∼4 fold higher in nAtf6 TG larvae compared to wildtype (WT) larvae (Figure 2A and S4) at 5 dpf, and was persistently elevated in older fish (Figure S4D and not shown). We detected *mCherry* mRNA in 5 dpf nAtf6 TG larvae by PCR, and Western blotting for mCherry detected the transgene in the liver of adult fish, albeit at much lower levels than achieved in transgenic fish expressing nuclear-localized mCherry without fusion to nAtf6 (i.e. *Tg(fabp10:nls-mCherry)*; [40] Figures S3D-E).

We confirmed that nAtf6 overexpression did not induce the other UPR branches by assessing *xbp1* splicing (Figure S4A) and Eif2a phosphorylation (Figure S4B) in the liver of nAtf6 TG 5 dpf larvae. We found no difference in these markers in unstressed nAtf6 TG and WT larvae, however, *xbp1* splicing was lower in transgenics than in WTs after exposure to stress (i.e. 1 µg/ml tunicamycin for 48 hours; Figure S4A). This suggests that nAtf6 overexpression adapts hepatocytes to withstand a robust, pharmacologically-induced ER stress.

We next examined the expression of UPR target genes in the liver of 5 dpf transgenic and control larvae. We found that *bip* (also called *hspa5*), a transcriptional target of Atf6, was elevated at the protein (Figure S4C) and mRNA levels (Figure 2A and S4D) in nAtf6 TG larvae. mRNA-Seq analysis comparing control larvae (*Tg(fabp10:nls-mCherry);* n = 2 pools of ∼40–50 livers each; see [40] and GEO dataset GSE52605), ethanol-treated (n = 2 pools of ∼40–50 livers each), and nAtf6 TG larvae (n = 1 pool of ∼40–50 livers) revealed 33 UPR target genes to be highly and significantly upregulated in nAtf6 TG livers (Tables S2-S4 and Figure 2A; GEO dataset GSE56498). These genes were identified as ATF6 target genes in other studies [16], [41] or by having the UPR or protein folding as a primary GO descriptor, and included ER chaperones (*bip/hspa5, dnajc3,* and *grp94/hsp90b1*), quality control effectors (*canx, calret, calrl,* and *calrl2*), protein disulfide isomerases (*pdia3, pdia4*) and ERAD components (*edem1, derl1* and *derl3*). A subset of these genes was confirmed by qPCR at 5 dpf and at 14 dpf (Figure 2A and S4D-E). *atf4* and *derl1* were the only genes detected as significantly upregulated by qPCR but not by mRNA-Seq.

Analysis of the mRNA-Seq data from ethanol-treated larvae revealed a striking overlap between the “UPR-ome” induced by ethanol and by nAtf6 overexpression: of the 49 UPR target genes induced by ethanol, 30 were also induced in the liver of nAtf6 TG larvae (Figure 2B and Table S2). It is possible that the remaining 19 genes unique to the ethanol-induced transcriptome are attributed to the transient, early increase in *xbp1* splicing [13] or to other transcription factors that regulate their expression. Collectively, these data indicate that Atf6 is a major regulator of the UPR in ALD.

### Atf6 overexpression causes steatosis

We next asked whether nAtf6 was sufficient to cause FLD (Figure 2C-E). Induction of the *fabp10:*nAtf6-cherry transgene occurs at ∼2.5 dpf [42] and by 4 dpf, 44% of nAtf6 TG larvae developed steatosis which increased to 69% and 76% by 5 and 5.5 dpf, respectively (Figure 2C-D and S5A) and persisted until at least 14 dpf (Figure S5A), but there were no marked histological abnormalities in the nAtf6 TG liver at 5 dpf (Figure S5B). Hepatic triglyceride (TAG) levels were nearly doubled at 5 dpf, and six times higher in 14 dpf nAtf6 TG larvae (Figure 2E), clutch-to-clutch variability non-withstanding. However, TAG levels were the same in WT and nAtf6 TG adults (Figure 2E), despite persistent expression of the transgene (Figure S3E and not shown). We speculate that, over time, animals adapt to nAtf6 overexpression or that other metabolic parameters affect hepatic lipid accumulation in adult fish. Together, our data demonstrate that Atf6 is necessary for alcoholic steatosis and sufficient to cause steatosis in the absence of any other stress.

### nAtf6 overexpression alters expression of genes involved in lipogenesis and lipid export

**Figure 3:**
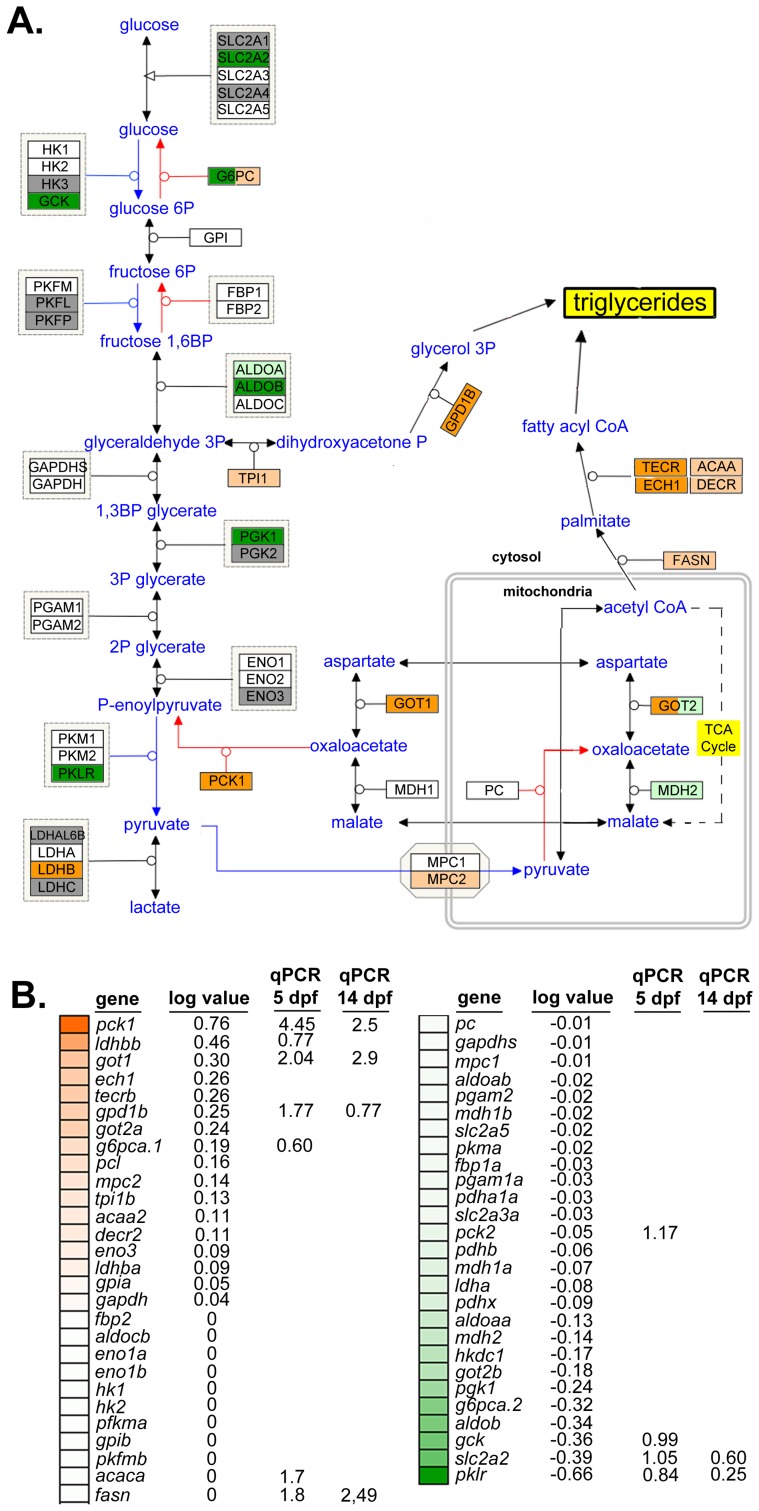
Glyceroneogenesis and fatty acid elongation pathways are dysregulated by nAtf6 overexpression. Schematic of the glycolysis, gluconeogenesis and glyceroneogenesis pathway (**A**, adapted from WikiPathways) and heatmap of associated genes (**B**). Upregulated genes are colored in shades of orange, downregulated genes are colored in shades of green, genes that are unchanged are white and those genes that did not appear in mRNA-Seq analyses are colored gray. Log values and median fold changes from mRNA-Seq analysis and qPCR, respectively, are noted.

Steatosis is caused by elevated TAGs in hepatocytes resulting from increased lipid synthesis or uptake, or from decreased lipid utilization or export. We therefore analyzed pathways relevant to these processes in the mRNA-Seq dataset from nAtf6 TG livers to determine if any were dysregulated. TAGs are generated from linking free fatty acids (FFAs) to a glycerol backbone, which is generated by conversion of dihydroxyacetone phosphate (DHAP) to glycerol-3-phosphate (G3P) by G3P dehydrogenase (GPD1) (see schematic in Figure 3A). We found genes that participate in TAG synthesis (Figure 3A-B) or export (Figure S6) were dysregulated in nAtf6 TG livers at 5 dpf, albeit not to the same degree as observed for the UPR target genes (Figure 2). DHAP is generated via glycolysis or by glyceroneogenesis, a pathway that has significant overlap with gluconeogenesis (see [43] and schematic in Figure 3A). mRNA-Seq analysis demonstrated that several genes involved in glycolysis were downregulated in nAtf6 TG livers including *aldob*, *glut2/slc2a2, gck*, *pgk1,* and *pklr* (Figure 3A-B). Genes promoting glyceroneogenesis, particularly *pck1* and *got1*, which generates oxaloacetate for conversion to phosphoenoylpyruvate by *pck1,* were upregulated, as was *gpd1b*, which drives glycerol formation. This suggests that nAtf6 overexpression increases factors regulating glyceroneogenesis preferentially over those that promote glycolysis (Figure 3A-B). In addition, *ech1* and *tecrb,* two genes responsible for fatty acid elongation in the mitochondria and ER, respectively, were induced in nAtf6 TG livers by mRNA-Seq (Figure 3A-B). Some of these genes were confirmed by qPCR analysis of nAtf6 TG livers, with the median fold change from qPCR on 3 to 6 clutches of livers from 5 and 14 dpf larvae depicted in Figure S7.

TAGs are assembled in the hepatocyte ER and secreted in association with lipoproteins that are required for transport in plasma. Several genes that regulate lipoprotein assembly and export, including *apom, apoeb, apoea*, *cetp* and *zgc:162608* and *zgc:194131,* two apolipoprotein-like genes, were downregulated in nAtf6 TG livers (Figure S7 and Table S3). *pdia2*, which interacts with MTP to transport lipids out of hepatocytes [44], was also downregulated. These data suggest that hepatic lipid transport may be impaired by nAtf6 overexpression. While these expression data do not capture the complex metabolic and post-translational regulation of the proteins encoded by these genes, they do suggest enhanced lipogenesis or suppressed lipoprotein export as potential mechanisms of steatosis in nAtf6 transgenics.

### *De novo* lipogenesis is enhanced by nAtf6 overexpression

**Figure 4:**
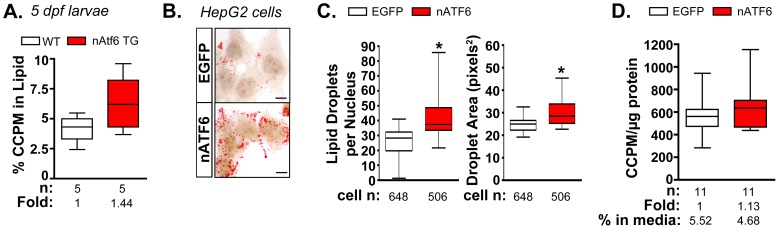
*De novo* lipogenesis is enhanced *in vitro* and *in vivo* by nAtf6 overexpression. **A:** ^14^C-acetate is preferentially incorporated into lipids in 5 dpf nAtf6 TG larvae compared to WT. The percent of ^14^C in lipid fraction was divided by ^14^C measured in the unextracted lysate from whole fish. The median fold change is noted; n =  number of samples of pooled larvae. **B:** Oil red O staining of HepG2 cells transfected with GFP or nATF6. Bar  = 10 µm. **C:** Quantification of lipid droplet number and area (square pixels). Statistics: unpaired t-test. *, p<0.05; n indicates the number of cells quantified over 20 fields from 2 independent experiments. **D:** Incorporation of ^14^C-acetate into lipids by HepG2 cells transfected with GFP or nATF6 normalized to protein. The median fold change is noted; n =  number of separate batches of cells analyzed in 2 independent experiments.

Acetate is a precursor to acetyl-CoA, the major building block of fatty acid and, therefore, TAG synthesis. To determine if nAtf6 overexpression increased *de novo* lipogenesis, we measured the incorporation of ^14^C-acetate into lipids in nAtf6 TG larvae (Figure 4A) and in HepG2 cells overexpressing nATF6 (Figure 4B-D) compared to their respective controls. Larvae were incubated with ^14^C-acetate from 3–5 dpf and we measured the amount of radiolabel present in the lipid fraction compared to that present in the lysate of the whole larvae. In an average of 5 individual pools of larvae, more ^14^C radiolabel was detected in the lipid fraction of nAtf6 TG larvae than in wildtype larvae (Figure 4A).

Since the liver in 5 dpf zebrafish larvae is too small to analyze incorporation in this organ exclusively, analysis of the whole larvae could not determine whether the label was preferentially incorporated into hepatocytes of nAtf6 TG larvae. We thus used human hepatoma (HepG2) cells transfected with human nATF6 to address this question. nATF6 overexpression causes accumulation of oil red O stained cytoplasmic droplets (Figure 4B) that are both greater in number and larger in size than in cells transfected with GFP (Figure 4B). These findings were confirmed in 293T cells (Figure S8) and correlate with our findings in nAtf6 TG larvae (Figure 2). Thus, nAtf6 overexpression causes lipid accumulation across cell types and species. Moreover, in eleven independent cell samples from two separate experiments, HepG2 cells overexpressing nATF6 had slightly more ^14^C radiolabel in the lipid fraction compared to GFP-transfected cells (Figure 4D). While we found that the incorporation tended to be higher in the nATF6 expressing cells, the average increase was moderate, at best. We attribute this to the transfection efficiency of HepG2 cells combined with experimental conditions used to label lipids, which likely act to select against nATF6 expressing cells.

### Srebp activation does not contribute to Atf6-mediated steatosis

**Figure 5:**
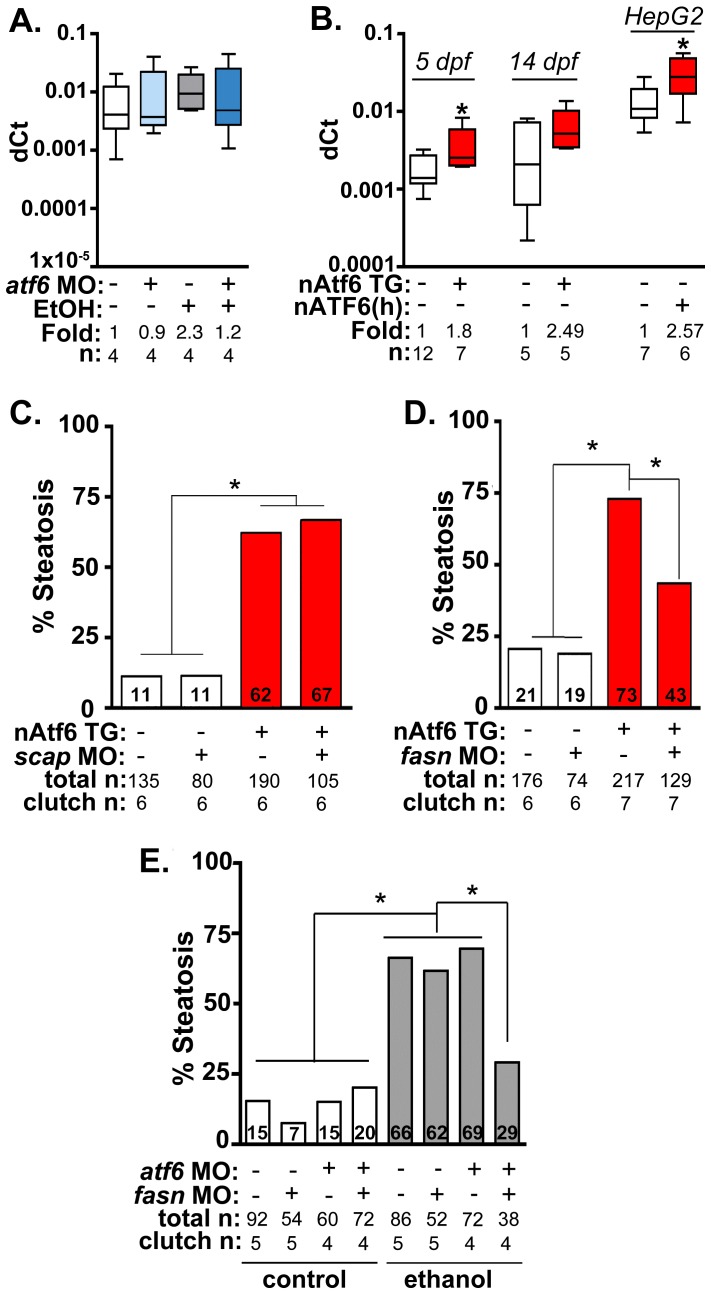
Fasn functions downstream of Atf6 to cause steatosis independent of Srebp activation. **A:** qPCR analysis of *fasn* expression in *atf6* morphants and uninjected larvae treated with EtOH for 32 hours. **B:** qPCR analysis of *fasn* expression in nAtf6 TG larvae and HepG2 cells transfected with nATF6. Statistics: unpaired t-test. *, p<0.05. **C:** Quantification of whole mount oil red O staining of nAtf6 TG larvae injected with *scap* MO. “Total n” and “clutch n” corresponds to the number of larvae and number of clutches scored, respectively. Statistics: chi-square with Fisher's Exact Test. *, p<0.05. **D:** Quantification of oil red O staining in nAtf6 TG larvae injected with *fasn* MO. **E:** Quantification of oil red O staining in *atf6/fasn* double morphants treated with 350 mM EtOH for 24 hours. Statistics: chi-square with Fisher's Exact Test. *, p<0.05.

Fatty acid synthase (*fasn)* mediates multiple steps in fatty acid synthesis that culminate in generation of palmitate from acetyl-CoA, a building block for TAG biosynthesis. *fasn* transcription in hepatocytes is primarily regulated by Srebp1c [45]–[47], and we previously demonstrated that *fasn* transcripts and other Srebp target genes are induced in zebrafish with ALD [12], [13]. Moreover, blocking Srebp activation by injecting a morpholino targeting the Srebp activating protein, Scap, reduced the incidence of alcoholic steatosis [12], [13]. Since Atf6 and Srebps are activated by similar mechanisms – they are both retained as inactive precursors in the ER and processed by the same Golgi-resident enzymes [33] – Srebp mediated lipogenesis has been proposed as a mechanism by which UPR activation causes FLD [27], [32]. We tested this using a genetic approach. First, we asked whether blocking Atf6 affected the induction of lipogenic gene expression in the liver of ethanol treated larvae. Both *fasn* (Figure 5A), and *acc1* (*acaca;* Figure S9A) were upregulated by ∼2 fold in the liver of ethanol treated larvae, and this was blocked by *atf6* MO injection (Figures 5A and S9A). However, induction of other Srebp target genes was not blocked by Atf6 depletion, including *srebp1* (Figure S9A) and the Srebp2 targets *hmgcra, hmgcs1* and *mvk* (Figure S9B). Since the entire Srebp target gene set was not uniformly affected by *atf6* loss, we conclude that Atf6 does not interact with Srebps. Instead, Atf6 may affect expression of specific lipogenic genes (i.e. *fasn* and *acc1*) by a mechanism distinct from Srebp1. Interestingly, we found mild induction of Srebp2 target genes in untreated *atf6* morphants (Figure S9B), supporting conclusions based on experiments in mammalian cultured cells where ATF6 was found to directly suppress SREBP2 activity [48].

Next, we asked whether nAtf6 was sufficient to induce *fasn* and *acc1* by assessing their expression in livers of nAtf6 TG larvae. Both *fasn* (p<0.05; Figure 5B) and *acc1* (Figure S9C) were increased by ∼2 fold at 5 dpf, and *fasn* expression was also higher at 14 dpf in nAtf6 TG larvae and in HepG2 cells overexpressing nATF6 (Figure 5B). However, other Srebp1 and Srebp2 genes were not significantly induced by nAtf6 overexpression in nAtf6 TG larvae (Figure S9C-D) or in HepG2 cells overexpressing human nATF6 (not shown), indicating that nAtf6 does not activate the entire Srebp target gene program. We then tested whether nAtf6 functioned upstream of Srebps by assessing whether *scap* morpholino injection suppressed steatosis in nAtf6 TG larvae. While *scap* morphants are resistant to ethanol-induced steatosis [12] we found no difference in steatosis incidence in nAtf6 transgenics (Figure 5B). Finally, to test whether *atf6* and *scap* interacted to cause alcoholic steatosis we injected low concentrations of morpholinos targeting both genes and found that they did not synergize to suppress alcoholic steatosis (Figure S10). We thus conclude that lipogenic gene induction and steatosis caused by nAtf6 overexpression does not require Srebps.

### Steatosis in nAtf6 TG larvae requires *fasn*

**Figure 6:**
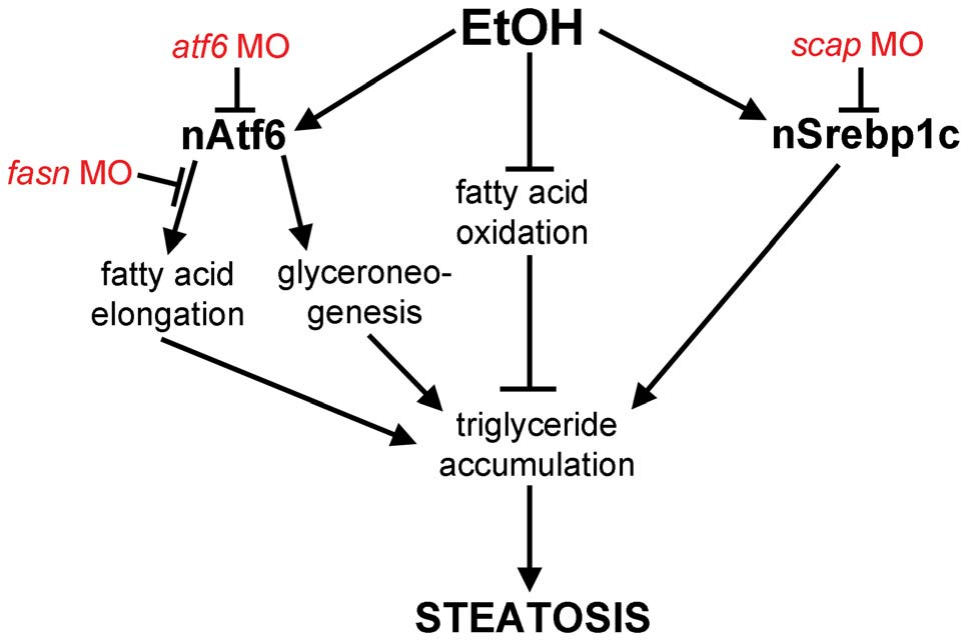
Working model by which Atf6 functions as a positive regulator of alcoholic steatosis. Genetic tools used in this study are illustrated in red.

The functional relevance of the finding that nAtf6 overexpression increases lipogenesis was tested by assessing the effect of morpholino-mediated *fasn* (Table S1) knockdown on steatosis incidence in nAtf6 TG larvae. Injection of high concentrations of *fasn* MO induced severe morphological defects, so we optimized the *fasn* MO concentration to have minimal toxicity. This had no effect on steatosis incidence in control larvae, but reduced steatosis incidence in nAtf6 TG larvae from 74% to 42% (Figure 5C). Furthermore, we found that *atf6* and *fasn* interacted epistatically to cause alcoholic steatosis by co-injecting concentrations of *fasn* and *atf6-*SPL MOs that did not have any effect on alcoholic steatosis on their own but, together, significantly reduced steatosis incidence in response to ethanol (Figure 5D). Thus, Atf6 and Fasn function in the same genetic pathway. Taken together, our data suggest a model by which Atf6 causes steatosis, in part, by inducing *fasn* and TAG synthesis by a mechanism that is independent of Srebps (Figure 6).

## Discussion

There is a wealth of recent literature showing that the UPR is activated in FLD and that this aspect of the disease is conserved across species [4], [16], [21], [23], [49]–[51]. However, it has not been shown that any single UPR factor can directly cause this disease and the mechanism by which UPR activation causes lipid accumulation is unclear. Here, we use zebrafish genetics and biochemical analyses to show that Atf6 is both necessary and sufficient for steatosis by showing that: **(i)** Atf6 is required for alcoholic steatosis, **(ii)** activation of Atf6 is sufficient to cause steatosis, **(iii)** Atf6 activation induces expression of genes involved in glyceroneogenesis and fatty acid elongation and causes *de novo* lipogenesis, and **(iv)** Atf6 epistatically interacts with fatty acid synthase (*fasn*), a key enzyme involved in TAG biosynthesis, to cause FLD. This is among the first data to directly link a main UPR sensor and lipid metabolic pathways. 

A number of studies have demonstrated that loss of one of the key UPR sensors – PERK [19], IRE1α [20] or ATF6 [16], [21], [23] – enhances steatosis caused by acute stress. Additionally, we previously demonstrated that Atf6 depletion suppresses steatosis caused by chronic stress [23]. Thus, it is clear that UPR activation is a conserved, common feature of FLD, but whether it functions to promote or prevent steatosis appears to depend on the nature and duration of the FLD-causing stress. Importantly, these loss of function studies have described a requirement for the UPR in FLD caused by tunicamycin injection, but not in the context of a stress that mirrors human conditions, such as obesity or alcohol abuse. Here, we show that Atf6 is induced in ALD, that its activation precedes steatosis, and that knocking down Atf6 reduces alcoholic steatosis. Thus, Atf6 is required for ALD. Moreover, since overexpression of nAtf6 is sufficient to cause FLD in the absence of any other stress, we conclude that this branch of the UPR is a pathophysiological mechanism of FLD.

Atf6 and Xbp1 are the primary transcription factors that both independently and cooperatively regulate expression of hundreds of UPR target genes. We previously found significant upregulation of many UPR target genes in zebrafish with ALD [11], [13] and hypothesized that since *xbp1* splicing in the liver was only an early, transient response to alcohol [13], other transcription factors must participate in this robust UPR. Transcriptome analysis of the livers from nAtf6 TG and ethanol treated larvae enabled us to identify a set of target genes that are likely to be directly activated by Atf6. Many of these genes were also reported to be upregulated in mice overexpressing inducible ATF6 in the heart [41] and they were not induced by tunicamycin treatment of *Atf6^-/-^* MEFs [16]. Genes occupying the intersection of these different datasets are likely *bona fide* Atf6 targets and the transgenic larvae we generated provides a system to test this directly. In contrast, many genes that were downregulated when ATF6 was overexpressed in the mouse heart were unchanged in nAtf6 transgenic zebrafish livers and, conversely, genes such as *canx, derl1,* and *atf4* were upregulated in the nAtf6 TG zebrafish liver samples but not in the mouse model. The variations between the two datasets may be due to inherent differences in the models or approaches used to detect changes in gene expression, or may reflect the ability of Atf6 to differentially regulate target genes depending on cell type: hepatocytes possess a significantly higher secretory capacity than cardiomyocytes, and thus the Atf6 transcriptome in hepatocytes may be more extensive in order to maintain ER homeostasis. Finally, comparative transcriptome analysis between nAtf6 TG and ethanol treated larvae identified significant overlap between the UPR target genes in these two datasets, indicating that Atf6 is the main transcriptional driver of the ethanol-induced “UPR-ome”.

What is the mechanism by which the UPR causes FLD? Our data showing that nAtf6 overexpression induces the expression of some lipogenic enzymes and increases lipid synthesis, and that blocking fatty acid synthesis reduces FLD, fits a model (Figure 6) whereby increased lipid production is, in part, the mechanism of steatosis in FLD caused by UPR activation. However, despite our finding that Atf6 depletion suppresses *fasn* expression and nAtf6 overexpression induces *fasn*, we have no evidence that *fasn* is a direct transcriptional target of Atf6. Indeed, the level of *fasn* induction is far less than the established Atf6 target, *bip*, and there are no canonical UPREs or ERSEs [15] in the *fasn* promoter (not shown), thus other pathways are also likely at play. The mechanism by which nAtf6 induces *fasn* and *acc1* require further investigation, and the tools we describe here will facilitate such studies.

The SREBP transcription factors are well-characterized regulators of hepatic lipogenesis; SREBP1c functions by increasing the expression of the full panel of genes required for TAG biogenesis [52]. One possibility, suggested by the finding that the UPR and SREBPs are activated in parallel in some systems [26], [27], [32], is that nAtf6 causes Srebp activation, leading to increased expression of *fasn* and other lipogenic genes and promoting lipogenesis. Our data argues against this: **(i)** the full panel of Srebp targets are not induced by nAtf6 overexpression in zebrafish, **(ii)** Atf6 and Srebps did not epistatically interact to modify alcoholic steatosis and **(iii)** Srebp activation is not required for steatosis in nAtf6 TG larvae. While it does appear that Srebp activation is required for alcoholic steatosis [12], [28], [29], the current study shows that it is not the only important pathway that regulates hepatic lipid metabolism, as steatosis in nAtf6 TG larvae is independent of Srebps.

While Srebps are not required for the effects of Atf6 on steatosis, there does appear to be some interaction between Atf6 and Srebp2, consistent with *in vitro* data showing that ATF6 suppresses SREBP2 [48]. We found that Atf6 causes upregulation of Srebp2 target genes, and while this modest increase in Srebp2 target genes in *atf6* morphants did not appear to have a functional impact on cholesterol biogenesis, since *atf6* morphants do not develop steatosis but are protected from it, it does suggest that Atf6 may suppress Srebp2 activity.

We used mRNA-Seq analyses to identify potential pathways that are dysregulated in response to Atf6 overexpression, although it is clear that the complex post-translational regulation of these pathways are not captured by gene expression studies. Our data suggested that lipoprotein export may be impaired by Atf6 overexpression, but we did not find that the flux of ^14^C-acetate into the extracellular lipids was impaired in cells overexpressing nATF6. While there are some technical caveats to these biochemical studies, we speculate that decreased lipoprotein export is not the only mechanism by which nAtf6 overexpression causes steatosis. Future studies to optimize cell conditions to ensure maximal cell viability and sustained high levels of nAtf6 expression will be required to fully address this.

While steatosis is clearly a first step on the continuum to more severe liver disease, we also propose a different possibility: that lipid accumulation is a sign of an active and adaptive stress response. Protein folding in the ER is metabolically demanding, and when secretory cargo increases or the ER becomes full of unfolded proteins, increased lipid accumulation may enhance the ATP supply and thereby sustain the increased protein folding demands. Thus, the UPR may serve to enhance the protein folding capacity of the ER function in two ways: by activating genes required for protein folding, quality and export, and by promoting metabolic flux to TAGs as an energy source to withstand the challenge presented by a high level of secretory cargo. In this scenario, acute steatosis would not be a pathological response but, instead, would provide a protective role to sustain hepatocyte function during stress. However, if the stress response persists and steatosis becomes chronic, lipotoxicity could potentiate liver injury. Based on these data, it is tempting to speculate that enhancing protein folding in the ER by chemical chaperones, which have been used in both mouse models and humans to alleviate UPR activity, attenuate fatty liver disease and increase insulin sensitivity [3], [53], [54], could reducr the demand for energy and thus reduce the need to accumulate lipid. Whether Atf6 functions both as sensor of unfolded proteins and of metabolic demand remains to be elucidated.

## Materials and Methods

### Animal husbandry, treatments and transgenics

Adult wildtype (WT, TAB14 and AB), *Tg(fabp10:dsRed*) [55] and *Tg(fabp10:nls-mCherry)* [40] zebrafish were maintained according to standard conditions. Larvae were exposed to 350 mM ethanol (Pharmco-AAPER, Brookfield, CT) in fish water starting at 96–98 hours post fertilization (hpf) for up to 32 hours as described [11], [12]. All zebrafish protocols were approved by Mount Sinai's Institutional Animal Care and Use Committee.

Morpholinos targeting the translation initiation ATG of *atf6* (*atf6*MO*-*ATG) [23], an *atf6* intron-exon boundary (*atf6*MO-SPL, Figure S1), a *scap* intron-exon boundary [12], and a *fasn* intron-exon boundary were ordered from GeneTools (Philomath, OR). Approximately 1–5 pmol were injected into 1–2 cell stage embryos. Morpholino sequences and amount injected per embryo (ng) are listed in Table S1.

The *Tg(fabp10:nAtf6-cherry; cmlc2:GFP)* transgenic line was created by injecting a vector containing 2813 bp of the *fabp10* promoter [55] upstream of the predicted nuclear fragment of zebrafish Atf6 (amino acids 1–366) as identified via DNA and protein alignments with human ATF6 (NCBI Reference Sequence: NP_031374.2). A cassette driving GFP expression in cardiomyocytes was included for rapid screening of transgenics (*cmlc2*:GFP). The transgene was flanked by Tol2 sites and the vector was injected into fertilized eggs along with transposase mRNA. Larvae were selected for *cmlc2:GFP* expression and raised to adulthood, outcrossed to TAB14 adults and 4 germline founders were identified.

### Plasmids

The nATF6-pcDNA-DEST47 plasmid was created using the Invitrogen Gateway System in which human nATF6 (amino acids 1–380) was amplified from a construct containing the full ORF (pEGFP-hATF6, from Dr. Aguirre-Ghiso) and ligated into pcDNA-DEST47. The nAtf6-GFP/pCI-Neo plasmid was generated by amplifying zebrafish nAtf6-GFP from a Tol2-generated plasmid (*fabp10:*nAtf6-GFP) with primers containing restriction sites for *Eco*RI and *Sma*I. The resulting PCR product was ligated into pCI-Neo via T4 ligase. pEGFP-C1 (Clontech) was used as a positive control for transfection.

### Cell culture and transfection

293T and HepG2 cells were grown in 100 mm dishes (BD Biosciences) with DMEM (Corning Cellgro, Manassas, VA) containing 10% fetal bovine serum (Invitrogen) and penicillin/streptomycin (Cellgro), and housed in a humidified incubator at 37 C with 5% CO_2_. Cells were passaged at 80–90% confluency either into 100 mm dishes or 6 well culture plates (Corning). Each well was transfected with 1–2 µg plasmid using Xtreme GENE 9 (Roche) or Lipofectamine 2000 (Invitrogen) for 24 hours. Following transfection, cells were collected for RNA extraction using TRIzol (Invitrogen), used for oil red O staining, or labeled with ^14^C-acetate as described below.

### ^14^C-acetate radiolabeling

HepG2 cells were washed with PBS 24–28 hours after transfection and kept in serum free high glucose DMEM (Corning Cellgro, Manassas, VA) containing penicillin/streptomycin (Cellgro), 100 nM insulin (Lilly USA, Indianapolis, IN) and 0.5 µCi ^14^C-acetate (PerkinElmer, Waltham, MA) for 17 hours. Media was harvested and spun to remove dead cells, while cells were scraped, washed twice and lysed in cold PBS. Protein concentration in the cell lysate was measured with a BCA protein quantification kit (Thermo Scientific, Waltham, MA). Fractions of the media and cell lysate portion were used for lipid extraction as described below.

WT and nATF6 5 dpf larvae were labeled with 1–5 µCi ^14^C-acetate for 48 hours. Larvae were washed twice and then sonicated in cold PBS. Protein concentration in the larval lysate was measured with a BCA protein quantification kit (Thermo Scientific). Fractions of the larval lysate were used for lipid extraction as described below.

### Lipid extractions

Lipids from media, cells and 5 dpf larvae were extracted according to Bligh and Dyer [56] with the following modifications. Briefly, methanol and chloroform were added to 400 µl media or 50 µl of 100 µl total cell lysate in a ratio of 2∶1. Samples were vortexed and chloroform and 0.45% NaCl were added at a ratio of 1∶1, vortexed again and phases were separated by centrifugation. The aqueous phase was re-extracted with one part chloroform and combined with the lipid from the first second extraction, washed with methanol and 0.45% NaCl at a 1∶1 ratio, centrifuged and the lipid phase was recovered and dried under a stream of N_2_ gas. Lipids were reconstituted in a 2∶1 mixture of chloroform and methanol (Thermo Scientific, Waltham, MA), dissolved in Ultima Gold and counted using a 2460 MicroBeta^2^ LumiJET (PerkinElmer, Waltham, MA) liquid scintillation counter. CCPM were normalized to protein concentration to account for variations in cell number.

### Quantitative real-time PCR (qPCR)

Total RNA isolated using TRIzol (Invitrogen) from pools of 15–20 dissected livers was reverse-transcribed using qScript cDNA SuperMix (Quanta Biosciences, Gaithersburg, MD). qPCR using A Light Cycler 480 (Roche) with PerfeCTa SYBRGreen FastMix (Quanta Biosciences) was used as previously described [11]. Values for target gene were normalized to reference gene *rpp0*, and dCts calculated using the comparative threshold method (dCt  = 2^−(Ct, gene – Ct, rpp0)^). Primer sequences are listed in Table S1.

### Triglyceride measurements

Approximately 20 livers from control and nAtf6 transgenic larvae were dissected and lysed in 0.5% Triton-X 100 and heated at 65°C for 5 minutes to inactivate hepatic lipases. Triglycerides were measured using the Infinity Triglyceride Liquid Stable Reagent (Thermo Fisher Scientific, Waltham, MA) following manufacturer's instructions, and were normalized to total protein concentration as determined by Bradford Assay (Bio-Rad, Hercules, CA).

### Oil red O staining and quantification

Larvae were fixed in 4% paraformaldehyde (PFA; Electron Microscopy Sciences, Hatfield, PA) overnight at 4°C, stained with oil red O, and scored for steatosis as previously described [35]. Cryosections were stained by immersing slides in increasing concentrations of propylene glycol (85%, 100%) for 10 minutes followed by an overnight incubation in oil red O (0.5% in propylene glycol, Polysciences, Warrington, PA). Excess oil red O was removed the next day by sequential washes in 100% and 85% propylene glycol for 5 minutes. Nuclei were counterstained with hematoxylin.

HepG2 and 293T cells were stained as previously described [57] and counterstained with hematoxylin. Oil red O droplet area and number were calculated using ImageJ. In brief, images were split into the red, green, and blue channels and the green channel was further processed for quantification as described, as oil red O has an excitation at 510 nm [58]. Following this, the background was subtracted from each image to ensure only counting of oil red O droplets. Droplets equal or greater than five square pixels were counted and area quantified. The values were copied into Microsoft Excel and the average droplet area and number of droplets per nucleus were counted for each field. Twenty independent fields from nATF6 and GFP-transfected HepG2 and 293T cells were imaged at 60x magnification.

### RNA sequencing (mRNA-Seq)

Total RNA was isolated using TRIzol (Invitrogen) from pools of ∼40–50 livers dissected on 5 dpf. Two clutches of *Tg(fabp10:nls-mCherry)* larvae that were either untreated (control) or treated with 350 mM ethanol for 24 hours and one clutch of nAtf6 TG larvae were used. polyA-tailed mRNA was selected using oligo-dT beads and then fragmented. cDNAs were created using random-hexamers and ligated with bar-coded adaptors compatible with HiSeq 2000. Single-end, 100 bp reads were sequenced at the Genomics Core of the Icahn School of Medicine at Mount Sinai. Custom-built software was used to map the reads to the zebrafish genome (Zv9/DanRer7) and estimate the coverage of each gene. Briefly, the reads were split into three 32 bp parts after trimming 2 bp at each end and mapped to the genome using a suffix-array based approach. The median of coverage across the transcript was used as an estimate of gene expression. The expression values were quantile normalized and log-ratios were calculated by comparing nAtf6 TGs to the average of the controls. Each ethanol treated sample was compared to its paired control (untreated siblings). Unique Gene Ontology terms (GO terms) were assigned to each gene by ranking the GO terms by relevance to the biology of the response, and using annotations from the human orthologues if the zebrafish annotations were lacking.

The distribution of expression values were plotted to identify the peak in the distribution, which is the level of noise in the system. The values were regularized by adding the noise to each gene's expression level before the log-ratios were calculated. This ensures that genes with low expression do not contribute to list of genes with large fold-changes. An absolute natural log ratio of 0.2 was used as the cutoff (known, non-responding genes are all below this threshold). The list of genes that show changes above this cutoff were analyzed for pathway enrichment using GO terms annotated as described above.

This data is available via the Gene Expression Omnibus (GEO) at accession number GSE56498. The controls for this dataset, untreated 5 dpf *Tg(fabp10:nls-mCherry),* also serve as controls in GEO dataset GSE52605 [40] and were included in both sets for ease of comparison between genotypes and treatments.

### Image processing and graphics

Images were cropped and minimally processed using Adobe Photoshop CS4 (Adobe Systems, San Jose, CA). Graphs were plotted using Prism 5.0c (GraphPad Software Inc., La Jolla, CA). Heat maps for mRNA-Seq data were generated using GENE-E (Broad Institute, Cambridge, MA). The global maximum and minimum for each gene set was set to orange and green, respectively, and zero was set to white. Metabolic pathway schematics were adapted from WikiPathways (URL: www.wikipathways.org).

### Statistical analyses

Statistical tests were performed using GraphPad QuickCalcs (GraphPad Software, URL: http://www.graphpad.com/quickcalcs/index.cfm). For oil red O staining of whole larvae, chi-square with Fisher's Exact test were performed. For qPCR, radiolabeling, and oil red O staining of HepG2 and 293T cells we performed unpaired and one-sample t-tests as appropriate.

### Supplementary methods

Methods for histological analysis, cryosectioning and staining, and liver circularity analysis are listed in Text S1.

## Supporting Information

Figure S1 Injection of an *atf6* splice-blocking morpholino induces mis-splicing of *atf6* mRNA. **A:** Schematic of boundary between intron 1 and exon 1 of zebrafish *atf6.* Exon sequence is shown in black and intron sequence is shown in blue, with the morpholino shown in red. The start codon is highlighted in yellow. **B:** Gel electrophoresis of *atf6* morphants at 2 dpf showing additional bands compared to uninjected controls. **C:** Sequencing results from *atf6* morphants displaying inclusion of intronic sequence (highlighted in blue). The predicted protein translation is shown, and early stop codons are noted as red asterisks.
(TIF)

Figure S2 Knockdown of *atf6* does not confer resistance to morphological defects from alcohol exposure. **A:** Oil red O staining of cryosections from uninjected and *atf6* morphant larvae treated with 0 or 350 mM ethanol for 32 hours. **B:** Live images of uninjected and *atf6* morphant *Tg(fabp10:dsRed)* larvae treated with 0 or 350 mM ethanol for 32 hours. **C:** Quantification of liver circularity, a measure of hepatomegaly, following ethanol treatment in uninjected and *atf6* morphant larvae. Livers were traced and circularity quantified in ImageJ.
(TIF)

Figure S3 *Tg(fabp10:nAtf6-cherry; cmlc2:GFP)* larvae express the nAtf6-cherry transgene despite a lack of visual fluorescence. **A:** Schematic of human ATF6 transactivation (TAD), DNA-binding (bZIP) and transmembrane (TM) domains, cleavage sites for S1P (MBTPS1) and S2P (MBTPS2), and protein alignment with zebrafish Atf6. Numbers in the diagram correspond to amino acid positions in human ATF6. Numbers at the cleavage sites in the protein alignment correspond to amino acid positions in zebrafish Atf6. **B:** Confirmation of nuclear localization of zebrafish nAtf6 by transfection of nAtf6-GFP/pCI-Neo into HepG2 cells. **C:** Live image of *Tg(fabp10:nAtf6-cherry; cmlc2:GFP)* larva at 5 dpf. **D:** Agarose gel electrophoresis showing expression of Cherry mRNA in nAtf6 TG but not WT larvae. *rpp0* was used to ensure PCR efficacy. **E:** Western blot visualization of Cherry protein in adult nAtf6 transgenic zebrafish liver tissue. *Tg(fabp10:nls-cherry)* adult liver protein was used as a positive control. Actin was used as a loading control.
(TIF)

Figure S4 Overexpression of nAtf6 induces robust transcription of UPR target genes but does not activate Ire1a or Perk**. A:** Agarose gel electrophoresis visualization of *xbp1* splicing in WT and nAtf6 TG larvae in the presence and absence of tunicamycin (Tm). The percentage of *xbp1* splicing was quantified by measuring band intensity in ImageJ. **B:** Western blot visualization of Eif2a phosphorylation in livers of 5 dpf WT and nAtf6 TG larvae. Tunicamycin (1 µg/mL, 3-5 dpf) treated WT larvae were used as a positive control for immunoblotting. Fold was calculated over 2-3 separate experiments as noted. **C:** Western blot visualization of Bip protein in livers of 5 dpf WT and nAtf6 TG larvae. Fold change (below blot) was calculated from 3 separate experiments. **D** and **E:** Quantitative real-time PCR of multiple UPR effectors in the livers of WT and nAtf6 TG larvae at 5 (**D**) and 14 (**E**) dpf. Statistics: unpaired t-test. *, p<0.05.
(TIF)

Figure S5 Lipid accumulation is evident in nAtf6 TG larvae in the absence of other hepatic injury. **A:** Staining of cryosections with oil red O showing significant steatosis in nAtf6 TG larvae at 5 and 14 dpf. **B:** Hematoxylin and eosin staining of nAtf6 TG and control larvae at 5 dpf. No other alterations in hepatic pathology were observed. The number of larvae used is indicated.
(TIF)

Figure S6 mRNA-Seq reveals potential disruptions in lipid transport by nAtf6 overexpression. Heatmap of genes associated with lipid binding and transport (**A**) and basic schematic of lipid synthesis and lipoprotein circulation (**B**, adapted from WikiPathways). Upregulated genes are colored in shades of orange, and downregulated genes are colored in shades of green. Genes that are unchanged are colored white. Genes that did not appear in mRNA-Seq analyses are colored gray in the schematic.
(TIF)

Figure S7 nAtf6 overexpression induces transcription of genes involved in glyceroneogenesis. Quantitative, real-time PCR analysis of gene expression from WT and nAtf6 TG larvae at 5 dpf (**A**) and 14 dpf (**B**). Statistics: unpaired t-test. *, p<0.05. For **(A)**, “n” corresponds to the number of clutches used and approximately 8-12 livers were dissected for RNA extraction. For (**B**), individual livers were dissected for analysis.
(TIF)

Figure S8 Overexpression of nATF6 in 293T cells drives lipid accumulation. **A:** Staining of GFP and nATF6 transfected cells with oil red O. **B:** Quantification of lipid droplet number and area (square pixels). Droplet number and area were calculated using ImageJ. Statistics: unpaired t-test. *, p<0.05. The number of cells used for quantification is noted.
(TIF)

Figure S9 Srebp1 and Srebp2 target genes are not dysregulated by nAtf6 overexpression. **A and B**: qPCR analysis of Srebp1 (**A**) and Srebp2 (**B**) target genes in atf6 morphants treated with 350 mM EtOH for 32 hours. Fold changes were calculated based on median dCt values. **C and D:** qPCR analysis of Srebp1 (**C**) and Srebp2 (**D**) target genes in *Tg(fabp10:nAtf6-cherry; cmlc2:GFP)* larvae at 5 dpf. Fold changes were calculated based on median dCt values, and “n” corresponds to the number of clutches used.
(TIF)

Figure S10 Atf6 and Srebps do not epistically interact to prevent alcoholic steatosis. Steatosis incidence in larvae injected with *atf6, scap,* or *atf6 + scap* MO. “Total n” corresponds to the number of larvae scored. “Clutch n” corresponds to the number of clutches analyzed. Statistics: chi-square with Fisher's Exact Test. *, p<0.05 and corresponds to groups significantly different from uninjected controls. b, p<0.05 and corresponds to ethanol-treated groups significantly different from uninjected, ethanol treated larvae.
(TIF)

Table S1 Morpholino and primer sequences. **A:** Morpholino sequences. Amount of morpholino injected is based on the assumption that 4 nl of MO is injected per embryo. **B:** Primer sequences used for conventional and quantitative, real-time PCR.
(DOCX)

Table S2 UPR effectors upregulated by nAtf6 and by ethanol. Livers from 2 clutches of *Tg(fabp10:nls-mCherry)* expressing larvae or from 1 clutch of nAtf6 TG larvae were dissected and pooled for RNA extraction and library preparation. Log values ≥ 0.2 are highlighted in red.
(XLSX)

Table S3 Upregulated and downregulated genes in the liver of nAtf6 TG 5 dpf larvae. **A:** Upregulated genes in the liver of nAtf6 TG 5 dpf zebrafish larvae. Livers from 2 clutches of *Tg(fabp10:nls-mCherry)* expressing larvae or from 1 clutch of nAtf6 TG larvae were dissected and pooled for RNA extraction and library preparation. UPR target genes are highlighted in pink. GO terms were assigned as described in the Materials and Methods. **B:** Downregulated genes in the liver of nAtf6 TG 5 dpf larvae. Genes regulating lipoprotein secretion are highlighted in orange.
(XLSX)

Table S4 Raw data from mRNA-Seq. Livers from 2 clutches of *Tg(fabp10:nls-mCherry)* expressing larvae or from 1 clutch of nAtf6 TG larvae were dissected and pooled for RNA extraction and library preparation. Genes are organized from highest to lowest log value for nAtf6 TG larvae.
(XLS)

Text S1 Supplementary methods.
(DOC)
