# Supplementary figures and images for: Activating Transcription Factor 6 Is Necessary and Sufficient for Alcoholic Fatty Liver Disease in Zebrafish
Source: PLoS Genet. 2014 May 29;10(5):e1004335. doi: 10.1371/journal.pgen.1004335 (PMC4038464; doi:10.1371/journal.pgen.1004335)

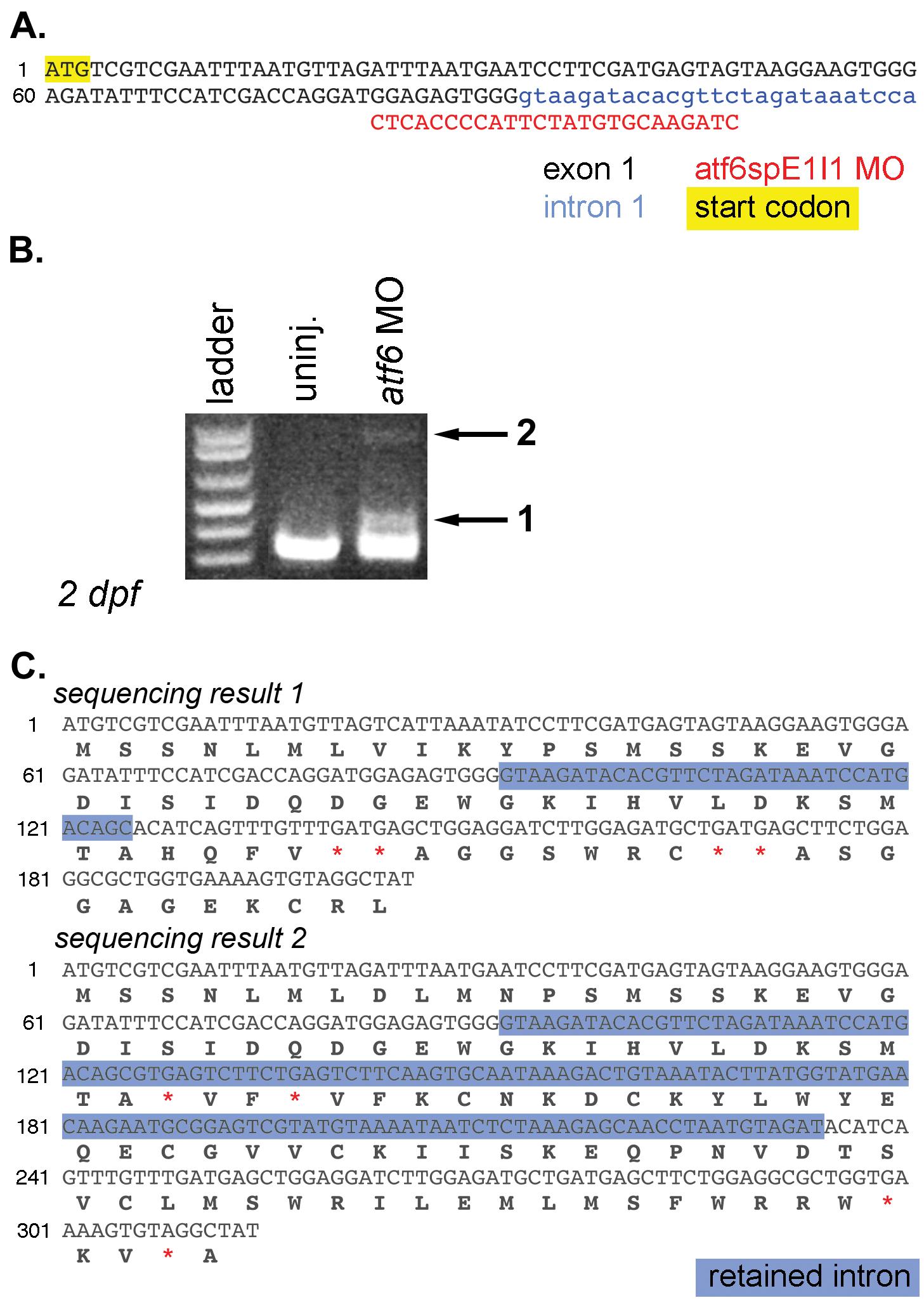

Supplement: Figure S1 — Injection of an atf6 splice-blocking morpholino induces mis-splicing of atf6 mRNA. A: Schematic of boundary between intron 1 and exon 1 of zebrafish atf6. Exon sequence is shown in black and intron sequence is shown in blue, with the morpholino shown in red. The start codon is highlighted in yellow. B: Gel electrophoresis of atf6 morphants at 2 dpf showing additional bands compared to uninjected controls. C: Sequencing results from atf6 morphants displaying inclusion of intronic sequence (highlighted in blue). The predicted protein translation is shown, and early stop codons are noted as red asterisks. (TIF) [file pgen.1004335.s001.tif]

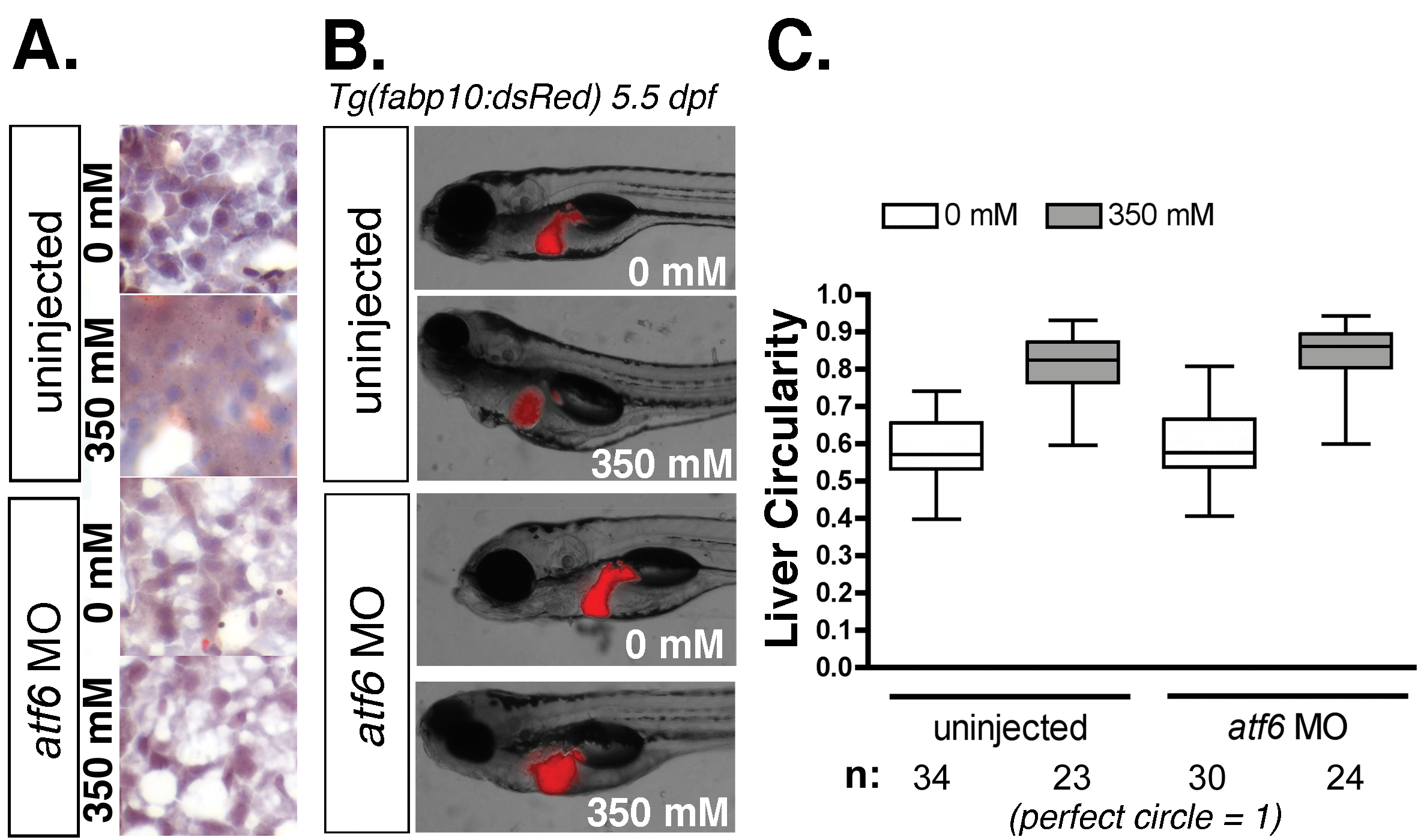

Supplement: Figure S2 — Knockdown of atf6 does not confer resistance to morphological defects from alcohol exposure. A: Oil red O staining of cryosections from uninjected and atf6 morphant larvae treated with 0 or 350 mM ethanol for 32 hours. B: Live images of uninjected and atf6 morphant Tg(fabp10:dsRed) larvae treated with 0 or 350 mM ethanol for 32 hours. C: Quantification of liver circularity, a measure of hepatomegaly, following ethanol treatment in uninjected and atf6 morphant larvae. Livers were traced and circularity quantified in ImageJ. (TIF) [file pgen.1004335.s002.tif]

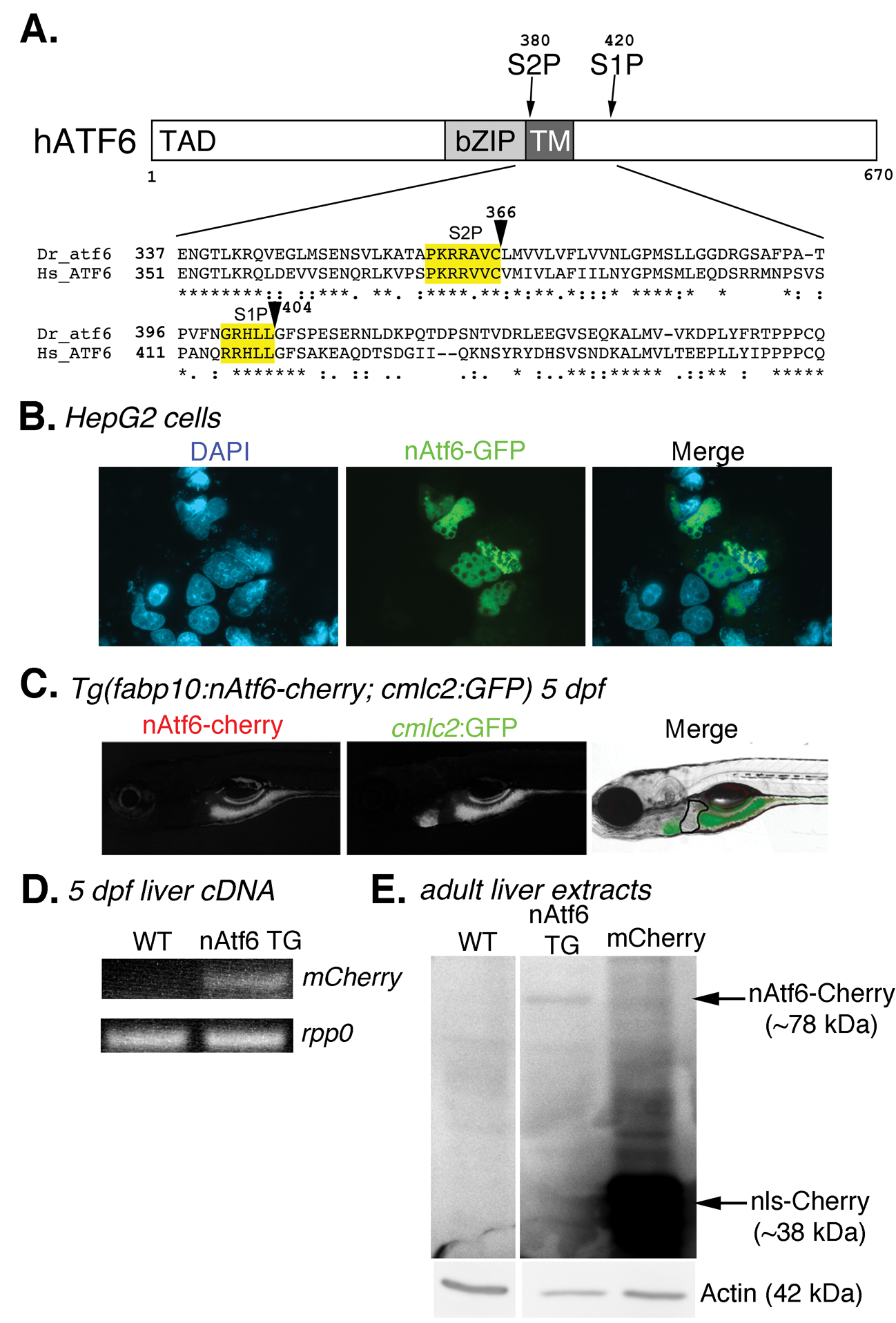

Supplement: Figure S3 — Tg(fabp10:nAtf6-cherry; cmlc2:GFP) larvae express the nAtf6-cherry transgene despite a lack of visual fluorescence. A: Schematic of human ATF6 transactivation (TAD), DNA-binding (bZIP) and transmembrane (TM) domains, cleavage sites for S1P (MBTPS1) and S2P (MBTPS2), and protein alignment with zebrafish Atf6. Numbers in the diagram correspond to amino acid positions in human ATF6. Numbers at the cleavage sites in the protein alignment correspond to amino acid positions in zebrafish Atf6. B: Confirmation of nuclear localization of zebrafish nAtf6 by transfection of nAtf6-GFP/pCI-Neo into HepG2 cells. C: Live image of Tg(fabp10:nAtf6-cherry; cmlc2:GFP) larva at 5 dpf. D: Agarose gel electrophoresis showing expression of Cherry mRNA in nAtf6 TG but not WT larvae. rpp0 was used to ensure PCR efficacy. E: Western blot visualization of Cherry protein in adult nAtf6 transgenic zebrafish liver tissue. Tg(fabp10:nls-cherry) adult liver protein was used as a positive control. Actin was used as a loading control. (TIF) [file pgen.1004335.s003.tif]

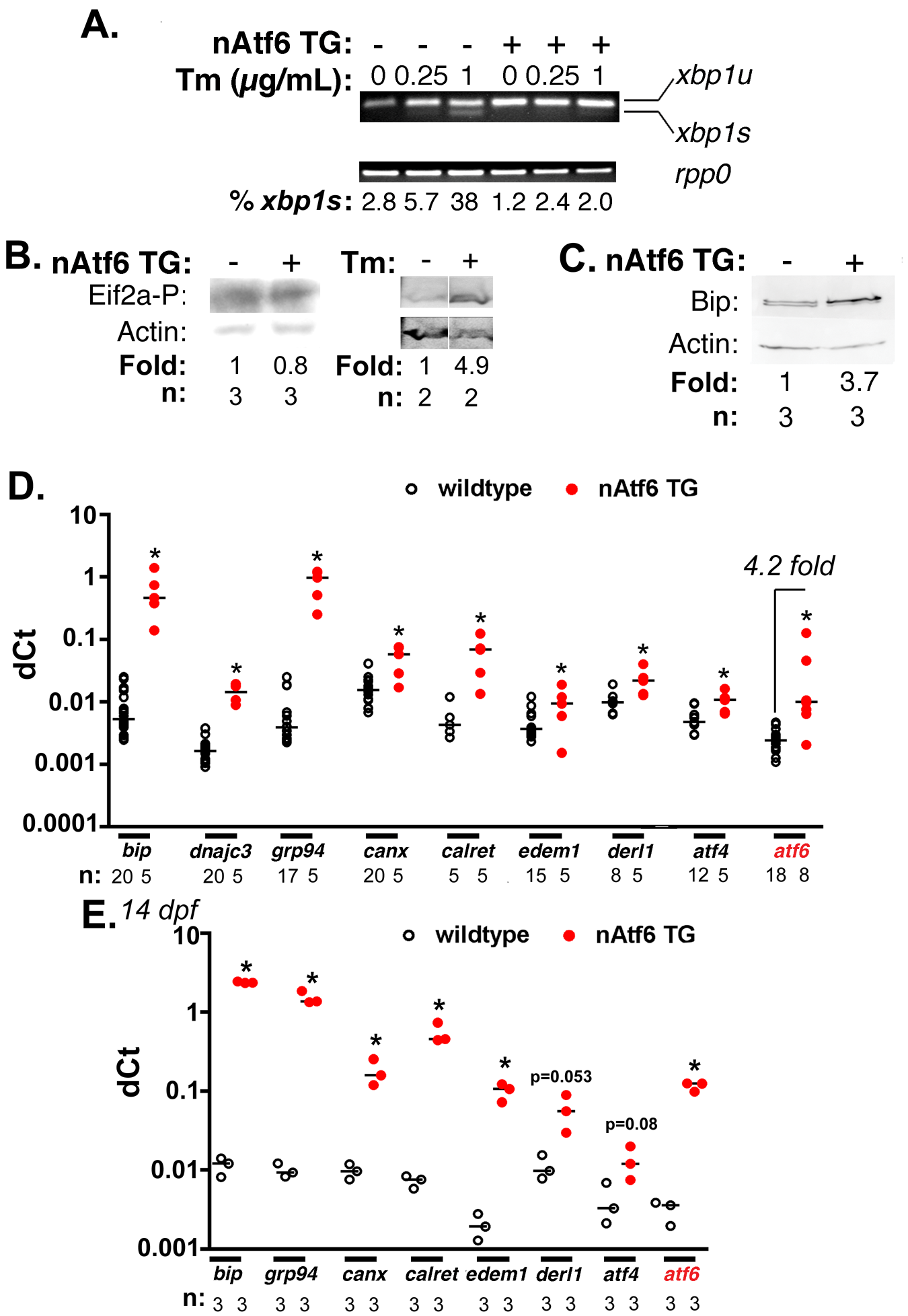

Supplement: Figure S4 — Overexpression of nAtf6 induces robust transcription of UPR target genes but does not activate Ire1a or Perk. A: Agarose gel electrophoresis visualization of xbp1 splicing in WT and nAtf6 TG larvae in the presence and absence of tunicamycin (Tm). The percentage of xbp1 splicing was quantified by measuring band intensity in ImageJ. B: Western blot visualization of Eif2a phosphorylation in livers of 5 dpf WT and nAtf6 TG larvae. Tunicamycin (1 µg/mL, 3-5 dpf) treated WT larvae were used as a positive control for immunoblotting. Fold was calculated over 2-3 separate experiments as noted. C: Western blot visualization of Bip protein in livers of 5 dpf WT and nAtf6 TG larvae. Fold change (below blot) was calculated from 3 separate experiments. D and E: Quantitative real-time PCR of multiple UPR effectors in the livers of WT and nAtf6 TG larvae at 5 (D) and 14 (E) dpf. Statistics: unpaired t-test. *, p<0.05. (TIF) [file pgen.1004335.s004.tif]

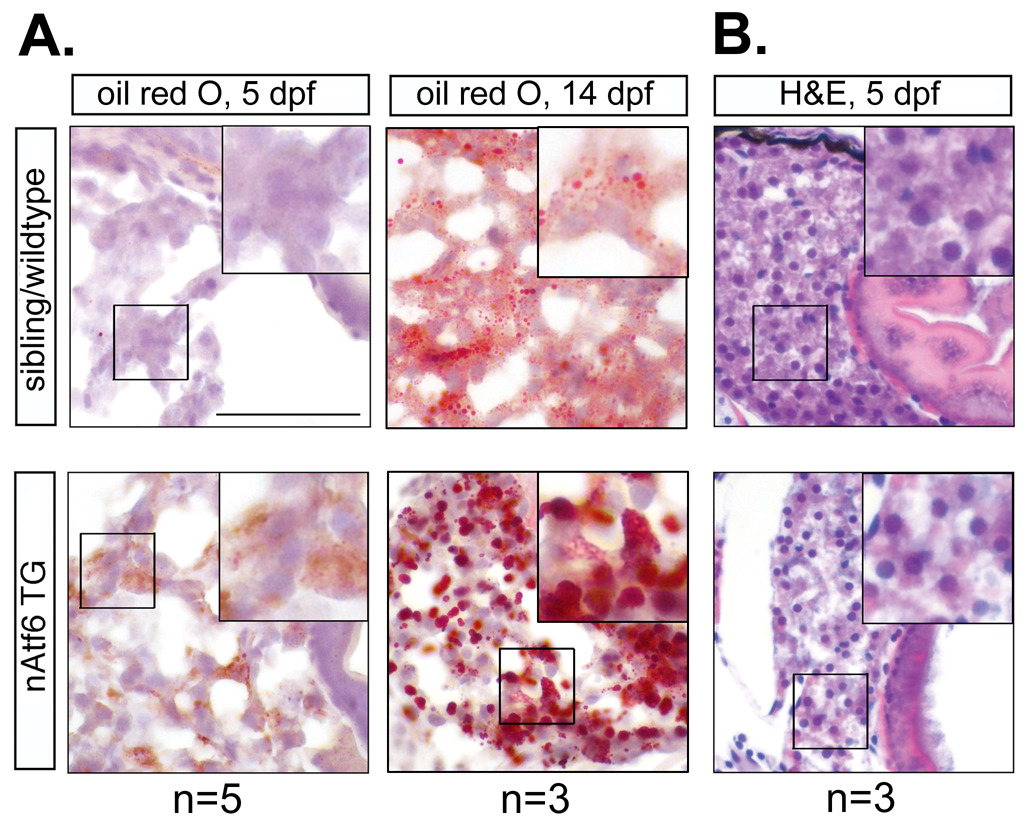

Supplement: Figure S5 — Lipid accumulation is evident in nAtf6 TG larvae in the absence of other hepatic injury. A: Staining of cryosections with oil red O showing significant steatosis in nAtf6 TG larvae at 5 and 14 dpf. B: Hematoxylin and eosin staining of nAtf6 TG and control larvae at 5 dpf. No other alterations in hepatic pathology were observed. The number of larvae used is indicated. (TIF) [file pgen.1004335.s005.tif]

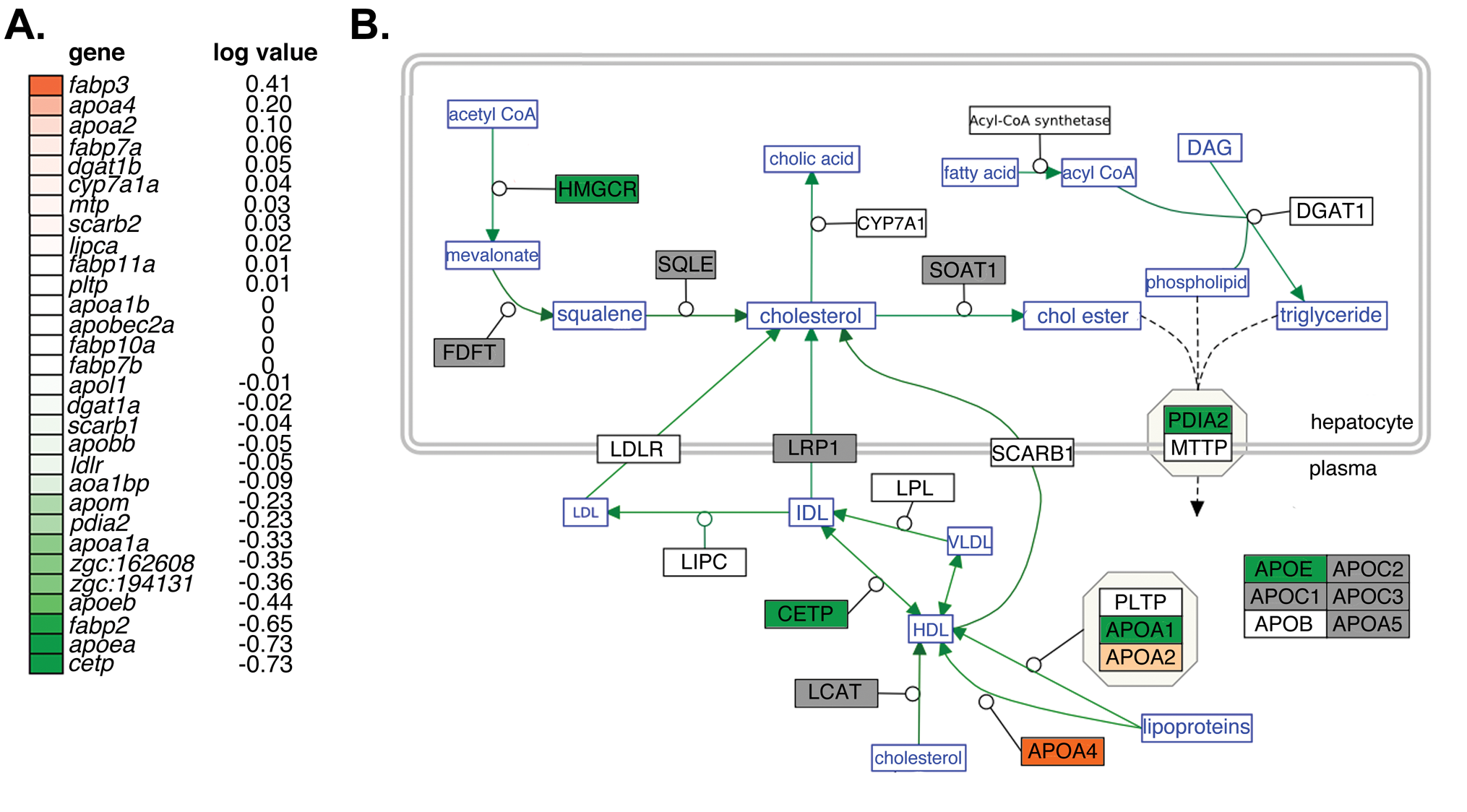

Supplement: Figure S6 — mRNA-Seq reveals potential disruptions in lipid transport by nAtf6 overexpression. Heatmap of genes associated with lipid binding and transport (A) and basic schematic of lipid synthesis and lipoprotein circulation (B, adapted from WikiPathways). Upregulated genes are colored in shades of orange, and downregulated genes are colored in shades of green. Genes that are unchanged are colored white. Genes that did not appear in mRNA-Seq analyses are colored gray in the schematic. (TIF) [file pgen.1004335.s006.tif]

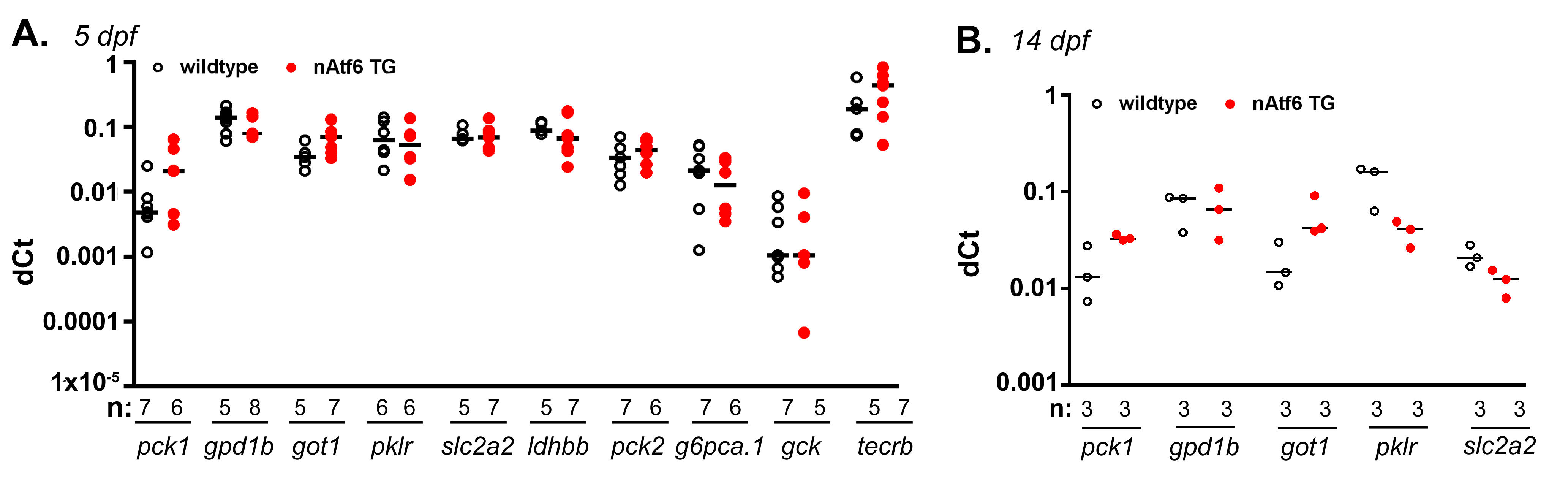

Supplement: Figure S7 — nAtf6 overexpression induces transcription of genes involved in glyceroneogenesis. Quantitative, real-time PCR analysis of gene expression from WT and nAtf6 TG larvae at 5 dpf (A) and 14 dpf (B). Statistics: unpaired t-test. *, p<0.05. For (A), “n” corresponds to the number of clutches used and approximately 8-12 livers were dissected for RNA extraction. For (B), individual livers were dissected for analysis. (TIF) [file pgen.1004335.s007.tif]

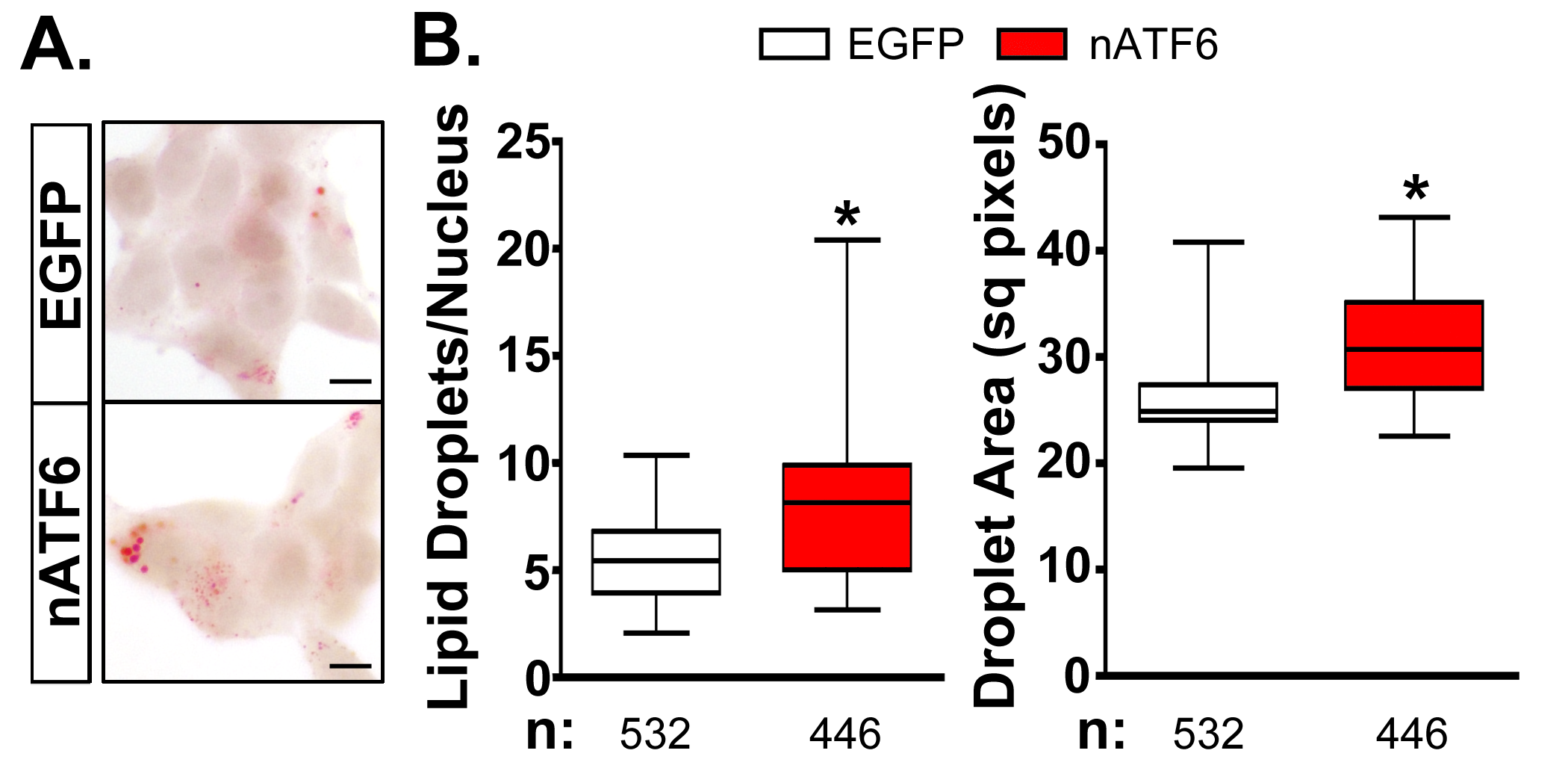

Supplement: Figure S8 — Overexpression of nATF6 in 293T cells drives lipid accumulation. A: Staining of GFP and nATF6 transfected cells with oil red O. B: Quantification of lipid droplet number and area (square pixels). Droplet number and area were calculated using ImageJ. Statistics: unpaired t-test. *, p<0.05. The number of cells used for quantification is noted. (TIF) [file pgen.1004335.s008.tif]

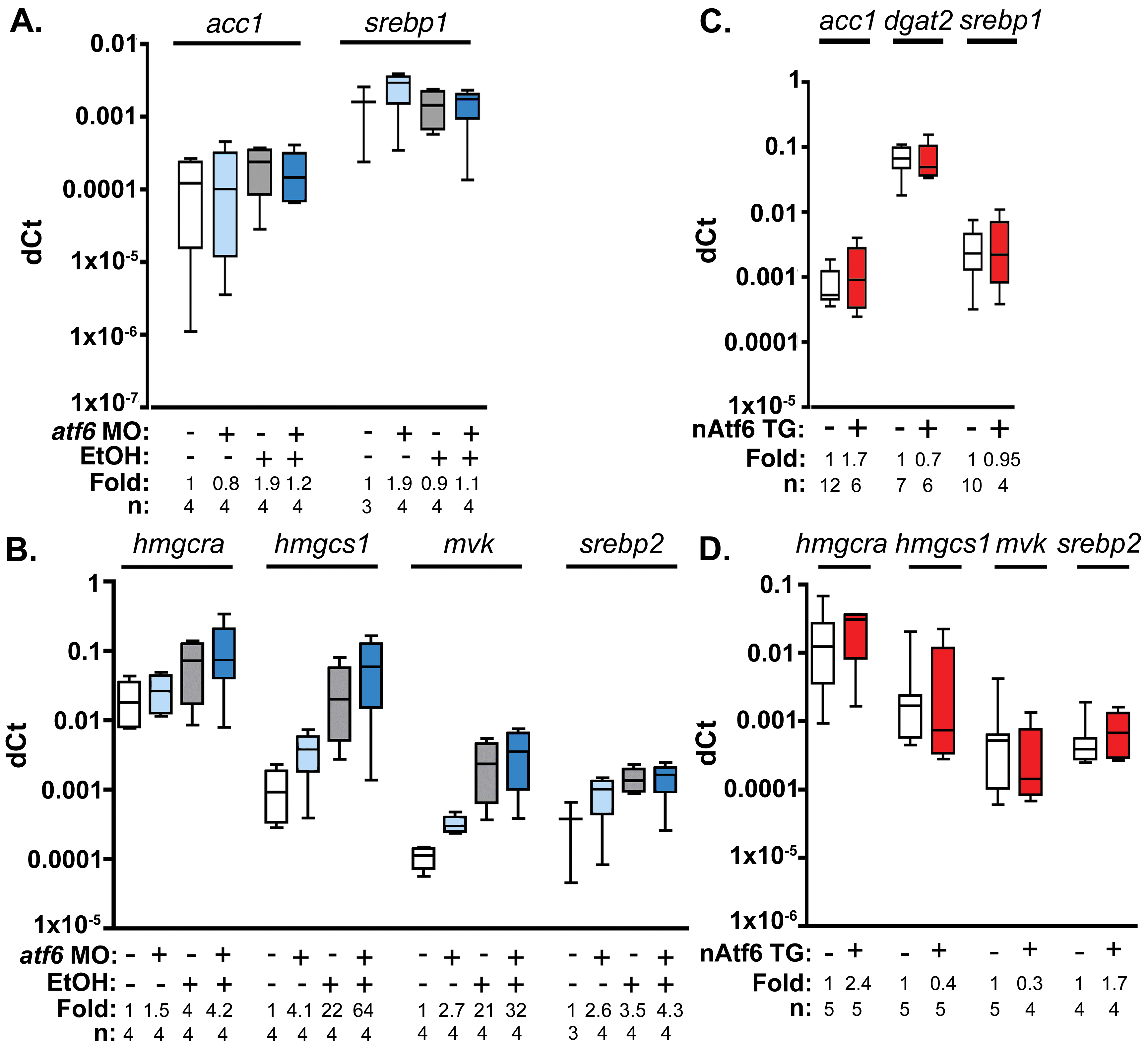

Supplement: Figure S9 — Srebp1 and Srebp2 target genes are not dysregulated by nAtf6 overexpression. A and B: qPCR analysis of Srebp1 (A) and Srebp2 (B) target genes in atf6 morphants treated with 350 mM EtOH for 32 hours. Fold changes were calculated based on median dCt values. C and D: qPCR analysis of Srebp1 (C) and Srebp2 (D) target genes in Tg(fabp10:nAtf6-cherry; cmlc2:GFP) larvae at 5 dpf. Fold changes were calculated based on median dCt values, and “n” corresponds to the number of clutches used. (TIF) [file pgen.1004335.s009.tif]

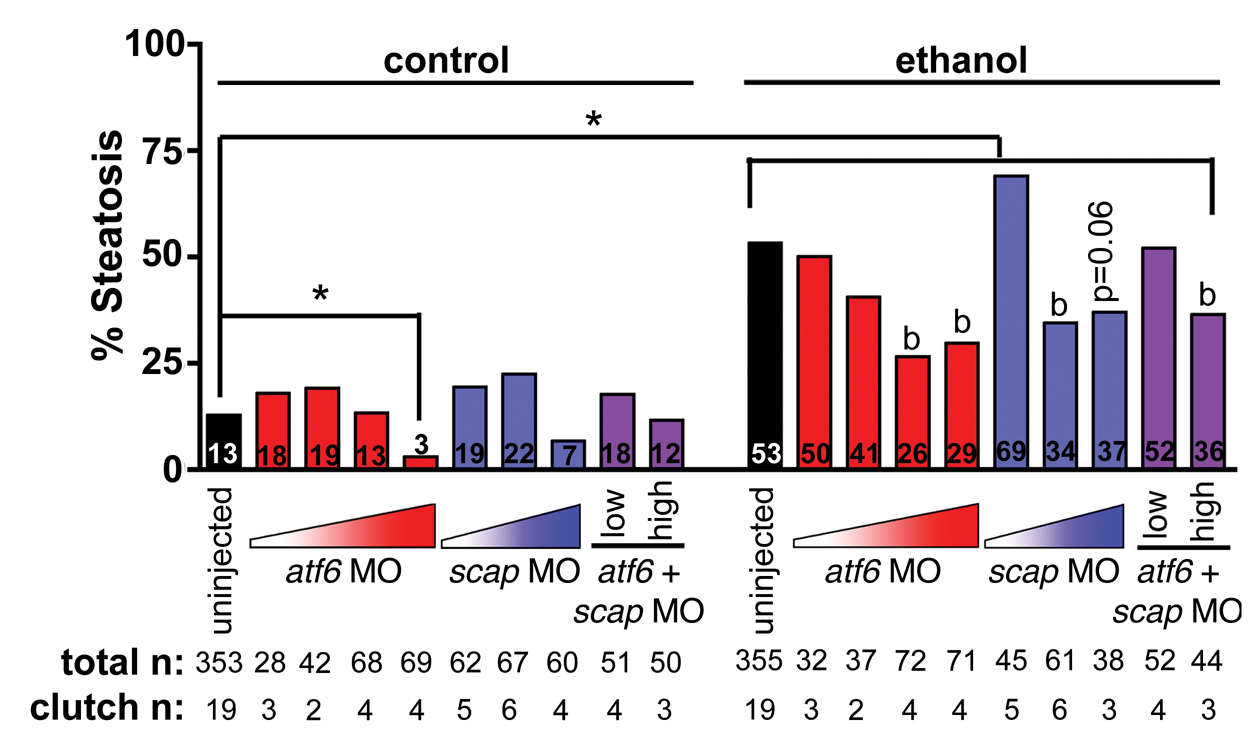

Supplement: Figure S10 — Atf6 and Srebps do not epistically interact to prevent alcoholic steatosis. Steatosis incidence in larvae injected with atf6, scap, or atf6 + scap MO. “Total n” corresponds to the number of larvae scored. “Clutch n” corresponds to the number of clutches analyzed. Statistics: chi-square with Fisher's Exact Test. *, p<0.05 and corresponds to groups significantly different from uninjected controls. b, p<0.05 and corresponds to ethanol-treated groups significantly different from uninjected, ethanol treated larvae. (TIF) [file pgen.1004335.s010.tif]
