# Supplementary material for: Activating Transcription Factor 6 Is Necessary and Sufficient for Alcoholic Fatty Liver Disease in Zebrafish
Source: PLoS Genet. 2014 May 29;10(5):e1004335. doi: 10.1371/journal.pgen.1004335 (PMC4038464; doi:10.1371/journal.pgen.1004335)
Supplement: Table S1 — Morpholino and primer sequences. A: Morpholino sequences. Amount of morpholino injected is based on the assumption that 4 nl of MO is injected per embryo. B: Primer sequences used for conventional and quantitative, real-time PCR. (DOCX) [file pgen.1004335.s011.docx]

**Table S1: Morpholino and primer sequences.**

**A:** Morpholino sequences. Amount of morpholino injected is based on the assumption that 4 nl of MO is injected per embryo.

| **Gene** | **Morpholino sequence (5’-3’)** | **Target** | **Amount Injected** |  |  |
| --- | --- | --- | --- | --- | --- |
| *atf6* | acattaaattcgacgacattgtgcc | ATG | 16.85 ng (0.5 mM) |  |  |
| *atf6* | CTAGAACGTGTATCTTACCCCACTC | intron-exon | 1.67-16.67 ng (0.05-0.5 mM) |  |  |
| *fasn* | GCGGTGGAAACCAGGTATGGCTGAC | intron-exon | 1.71-3.43 ng (0.05-0.1 mM) |  |  |
| *scap* | CCCGATACTGCAAGAAGATT | intron-exon | 2.71-13.6 ng (0.1-0.5 mM) |  |  |

**B:** Primer sequences used for conventional and quantitative, real-time PCR.

| **Gene name** | **Forward primer** | **Reverse primer** |
| --- | --- | --- |
| *acaca/acc1* | GCAAGTGTGGTTCCCTGATT | TCATGAAGGTCAGCGAACTG |
| *atf4* | TTAGCGATTGCTCCGATAGC | GCTGCGGTTTTATTCTGCTC |
| *atf6* | CTGTGGTGAAACCTCCACCT | CATGGTGACCACAGGAGATG |
| *bip/hspa5* | atcagatctggccaaaatgc | ccacgtatgacggagtgatg |
| *calret* | CAGTGTGCTTTATTTCTGCACTG | TAAAACCGAGCATCTTGACTTGT |
| *canx* | catctctgccctcctactgc | tccacaaactcctccaggtc |
| *cherry* | CCTGTCCCCTCAGTTCATGT | CCCATGGTCTTCTTCTGCAT |
| *chop* | atatactgggctccgacacg | gatgaggtgttctccgtggt |
| *derl1* | TTTCGACTTGGTGACGACTG | CAGAGCCCTCGTCACTTTTC |
| *dgat2* | catggcatcttgtgttttgg | tcggtttactgggcagattc |
| *dnajc3* | tcccatggatcctgagagtc | ctcctgtgtgtgaggggtct |
| *edem1* | gacagcagaaaccctcaagc | catggccctcatcttgactt |
| *fasn (zf)* | GAGAAAGCTTGCCAAACAGG | GAGGGTCTTGCAGGAGACAG |
| *FASN (human)*  *g6pca.1* | agtacacacccaaggccaag  tcacagcgttgctttcaatc | gtggatgatgctgatgatgg  ccacagatcacttggtgtgg |
| *gck* | gttggtgatttcctggcact | agccggcaatgtaatcaaac |
| *got1*  *gpd1b*  *grp94/hsp90b1* | aaccacaatgccgttttctc  aatgcaaacgcaaaacttcc  agcaagaccgagaccgtaga | atctgcttccattggtcctg  cggacttgctgtgcagaata  ctcccaatcccacacagtct |
| *hmgcra* | CTGAGGCTCTGGTGGACGTG | cgccGCAGCTACGATGTTGGcG |
| *hmgcs1* | ctcactcgtgtggacgagaa | gatacggggcatcttcttga |
| *ire1a* | TGACGTGGTGGAAGTTGGTA | ACGGATCACATTGGGATGTT |
| *mvk* | cacacttttgcgaccagaga | cagcagcaatgagcatctgt |
| *perk* | TGGGCTCTGAAGAGTTCGAT | TGTGAGCCTTCTCCGTCTTT |
| *pck1* | aactcactgctggggaagaa | gtctcccacacactccacct |
| *pck2* | acagactggcatgggaaatc | acacaccatgacgccagtta |
| *pklr* | tcctggagcatctgtgtctg | ctgatgagtgccgtgagaga |
| *rpp0* | ctgaacatctcgcccttctc | tagccgatctgcagacacac |
| *slc2a2*  *srebp1* | gcatttcaaggcgcttactc  actcttctggtgtggctgct | taagggaaaaccatgccaac  gagccttcagacacgtcctc |
| *srebp2* | cactcacacaagcacacacg | acctggttctggatgaatcg |
| *tecrb*  *xbp1* | ttcctgccccaattacacat  GGGTTGGATACCTTGGAAA | ctacaggatgaagggcagga  AGGGCCAGGGCTGTGAGTA |
