# Supplementary material for: Activating Transcription Factor 6 Is Necessary and Sufficient for Alcoholic Fatty Liver Disease in Zebrafish
Source: PLoS Genet. 2014 May 29;10(5):e1004335. doi: 10.1371/journal.pgen.1004335 (PMC4038464; doi:10.1371/journal.pgen.1004335)
Supplement: Text S1 — Supplementary methods. (DOC) [file pgen.1004335.s015.doc]

***Supplementary Information***

***Supplementary Materials and Methods***

**Tunicamycin treatments**

Larvae (wildtype or nAtf6 TG) were treated from 3-5 dpf with 0.25 or 1 μg/ml tunicamycin (Tm) for 48 hours as described [23]. DMSO was used as a solvent control.

**Histological analysis**

Cryosections were prepared as described [1]. Paraffin embedment was carried out as described [1,2]. Sections were cut at 4 µm and stained with hematoxylin and eosin by the Histology Shared Resource Facility at the Icahn School of Medicine at Mount Sinai.

**Liver circularity analysis**

Livers (left lobe) of control and ethanol treated *Tg(fabp10:dsRed)* larvae were imaged live using an RFP fluorescence filter. Livers were traced and circularity quantified in ImageJ. Circularity was used as a surrogate for left liver lobe enlargement.

***Supplementary References***

1. Imrie D, Sadler KC (2010) White adipose tissue development in zebrafish is regulated by both developmental time and fish size. Dev Dyn 239: 3013-3023.

2. Howarth DL, Yin C, Yeh K, Sadler KC (2013) Defining hepatic dysfunction parameters in two models of fatty liver disease in zebrafish larvae. Zebrafish 10: 199-210.
